# First Aid Curriculum for Second Year Medical Students

**DOI:** 10.21980/J8FH2J

**Published:** 2024-07-31

**Authors:** Megan Stodola, Megan Lantz, Tina Chen, Alexander Marelich, Isaac Philip

**Affiliations:** * Saint Louis University School of Medicine, St. Louis, MO; ^ Saint Louis University Hospital, Department of Emergency Medicine, St. Louis, MO

## Abstract

**Audience:**

This small-group workshop is designed for pre-clinical medical students. The workshop can also be offered to other medical students looking to review first aid in the community setting.

**Introduction:**

First aid training in medical students varies based on each student’s previous experience. Because of this, medical students in their pre-clinical years have expressed a desire for further training in first aid.^1^ While most bystanders in an emergency situation do not have a medical background, medical students have received additional training that can provide the skillset to process and respond to emergency situations in a different capacity. Most medical schools have not adopted a universal curriculum in teaching medical students first aid.^2^ Incorporating first aid into a medical school curriculum can enhance medical students’ confidence in emergent situations and lead to better outcomes for patients requiring immediate on-site care.

**Educational Objectives:**

The goal of this workshop was to improve the confidence of medical students in handling emergencies in the community with the use of first aid while also giving them a standard approach to emergencies using an airway, breathing, and circulation approach. The curriculum was evaluated through student-perceived self-efficacy and confidence in handling the provided scenarios, performance on relevant multiple choice questions, and general appeal of the first aid sessions. By the end of this workshop, students will be able to define the goals of “first aid” and first responder actions, describe clinical signs and symptoms suggestive of an airway, breathing, or circulation emergency in the setting of selected medical emergencies, and demonstrate immediate care steps in the setting of selected medical emergencies, specifically the Heimlich maneuver on adults and infants, direct pressure, wound packing, tourniquet application for external bleeding, epinephrine auto-injector administration, and the recovery position for obtunded or unconscious patients.

**Educational Methods:**

Small group activities were performed with a focus on case-based scenarios combined with hands-on instruction. The four scenarios were choking, seizure, anaphylaxis, and bleeding which were taught by an educator who was either faculty, an emergency medicine resident, or an upper-level medical student. Facilitators were provided an educational handout specific to their station to guide them through the teaching session. A PowerPoint presentation was also provided complete with supporting images and videos to share with the students each session.

**Research Methods:**

Students were asked to complete a pre-test and post-test survey to assess knowledge outcome, self-efficacy in first aid, and overall appeal of the workshop. The multiple-choice knowledge outcome data was scored for percent correct on each question as well as overall performance on questions grouped by content. Students were also asked to provide feedback and comments on their overall experience in the workshop.

**Results:**

Overall, medical students reported increased knowledge and confidence in responding to various first-aid situations. There was overall improvement in pre-test and post-test evaluations. The appeal of the event as a whole and its usefulness was overwhelmingly viewed as positive. Some participants noted they wanted similar workshops with more first-aid topics. Participants also noted they felt better prepared to respond to the various emergencies included in the workshop.

**Discussion:**

A workshop directed at teaching first aid to medical students increased their confidence and knowledge in responding to various emergencies and can successfully be accomplished through a focused large group didactic session and multiple clinically relevant small group teaching sessions.

**Topics:**

First aid, airway, breathing, circulation, medical students, choking, seizures, bleeding, anaphylaxis.

## USER GUIDE

[Table t3-9-3-sg63]

**Table t3-9-3-sg63:** 

List of Resources:	
Abstract	63
User Guide	65
Small Groups Learning Materials	69
Appendix A: Pre-curriculum Questionnaire	69
Appendix B: PowerPoint for Small Group Stations	73
Appendix C: Choking/Heimlich Maneuver for Adults, Children, and Infants	74
Appendix D: Seizure/Recovery Position	80
Appendix E: Anaphylaxis/Epinephrine Pen Injection	85
Appendix F: Bleeding/Pressure, Packing, Tourniquet	90
Appendix G: Post-curriculum Questionnaire	96
Appendix H: Multiple Choice Answer Key	101

**Learner Audience:**

Medical Students

**Time Required for Implementation:**

120 minutes

**Recommended Number of Learners per Instructor:**

5–6 students

**Topics:**

First aid, airway, breathing, circulation, medical students, choking, seizures, bleeding, anaphylaxis.

**Objectives:**

The goal of this curriculum is to:

1. Provide novice clinicians with the tools to recognize airway, breathing, or circulation life threats through small group facilitator-guided interactive discussion
2. Train novice clinicians in immediate, on-site responses to selected medical emergencies
3. Provide practice opportunities for selected first aid interventions in a low-fidelity, psychologically safe environment

By the end of this curriculum, participants will be able to:

4. Define the goals of “first aid” or first responder actions
5. Describe clinical signs and symptoms suggestive of an airway, breathing, or circulation emergency in the setting of selected medical emergencies
6. Demonstrate immediate care steps in the setting of selected medical emergencies, specifically:
  - The Heimlich maneuver on adults and infants
  - The recovery position for obtunded or unconscious patients
  - Epinephrine auto-injector administration
  - Direct pressure, wound packing, and tourniquet application for external bleeding

### Linked objectives and methods

This curriculum emphasizes developing the skills to recognize a patient in distress and deliver immediate, on-site first aid. Through these small groups, learners have the opportunity to practice these skills and get immediate feedback from experienced facilitators. Interactive small groups are superior to lectures in developing knowledge, skills, and attitudes. During small group sessions appendices C through F, students review airway, breathing, and circulation management in regards to each emergency First Aid response, meeting goals/objectives 1,4, and 5. In these same small groups, students participate in interactive educational question-based sessions and receive hands-on practice with emergency, on-site responses, meeting goals 2 and 3. During small group session appendix C on choking, students practice the Heimlich maneuver on manikins, meeting the first bullet point in objective 6. During small group session appendix D on seizures, students practice placing each other in the recovery position, meeting the second bullet point in objective 6. During small group session appendix E on anaphylaxis, students practice using the epinephrine auto injector, meeting the third bullet point in objective 6. During small group session appendix F on bleeding, students practice tourniquet application, meeting the fourth bullet point in objective 6.

### Recommended pre-reading for facilitator

See individual station guides

### Learner responsible content (LRC)

Thim T, Krarup NH, Grove EL, Rohde CV, Løfgren B. Initial assessment and treatment with the Airway, Breathing, Circulation, Disability, Exposure (ABCDE) approach. *Int J Gen Med*. 2012;5:117–121. At: doi:10.2147/IJGM.S28478

### Results and tips for successful implementation

The educational first aid simulation was required among second-year medical students at a time in the year when they had completed most of their preclinical courses. This was tested on 179 learners and 152 of those learners completed the post-test.

Learners were evaluated on their first aid knowledge as well as surveyed on their comfort in recognizing and providing care in several first-aid scenarios through a pre-test and post-test. The learners were also evaluated on their satisfaction with the curriculum through a survey in the post-test. Both self-efficacy and satisfaction surveys were conducted on a Likert scale where 1=strongly disagree, 2=disagree, 3=neither agree or disagree, 4=agree, and 5=strongly agree. Each student created a student identifier that was used when taking the pre- and post-tests. There were 14 exam questions. Pre and post-test overall exam scores as well as individual scores were evaluated with a student’s paired t-test with an alpha level of 0.05. The pre- and post-test competence survey was also evaluated with a student’s paired t-test with an alpha level of 0.05. The post-test satisfaction survey results were averaged among all students for each question.

The data showed a substantial improvement in student confidence and self-efficacy from the pre- and post-simulation surveys. Students’ performance on the multiple-choice questions also improved greatly demonstrating a better understanding and preparedness for first aid response. Interpretation of the subjective comments supports a general appeal of the sessions with suggestions for improvements. Future courses could incorporate the material into the first year of medical school with a refresher course in year two and decrease repetitiveness in content from other preclinical courses. The simulation should also emphasize as much hands-on practice as possible. While no notable immediate modifications were made, these modifications could be implemented in future sessions to improve the flow and educational experience. The course should continue to consist of small group sizes, maintain the twenty minutes per session flow, and have strong facilitators.

This data can be generalized to others who work in the healthcare field since that subset of people has more medical training than the general public, allowing them to approach a medical emergency in the context of airway, breathing, and circulation. Limitations of the study include the lack of participant response on the post-test, the loss of one small group teaching session due to a scheduling miscommunication, and the use of alternating facilitators for each station. The data supports that this group of second-year medical students left the first aid simulation sessions with a stronger knowledge base than they had when they entered and had a positive experience at the sessions overall.[Table t1-9-3-sg63][Table t2-9-3-sg63]

### Associated Content

Pre Session:

Appendix A: Pre-curriculum Questionnaire

Stations:

Appendix B: PowerPoint for Small Group Stations

For individual stations:

1. Appendix C: Choking/Heimlich Maneuver for Adults, Children, and Infants
2. Appendix D: Seizure/Recovery Position
3. Appendix E: Anaphylaxis /Epinephrine Pen Onjection
4. Appendix F: Bleeding/Pressure, Packing, Tourniquet

Post Session :

Appendix G: Post-curriculum Questionnaire
Appendix H: Multiple Choice Answer Key

### Pearls

What is First Aid?

- First Aid is the immediate medical care for an injured or ill person that a person can take until more advanced medical personnel arrive.^3^

What are the Signs of an Airway, Breathing, and Circulation Issue?

- An airway issue can be identified as a change in phonations, increased secretions, presence of vomit or blood, or noisy breathing.^4^
- A breathing issue can be identified through a change in a person’s work of breathing, changes in their chest rise or any obvious chest wall deformity.^4^
- A circulation issue can be identified as changes to skin color or warmth, changes in capillary refill, mental status changes, and obvious signs of hemorrhage.^4^

Things to Remember for Choking

- Signs of choking include the universal signal for choking, inability to speak, coughing, labored breathing, and changes in coloration.^5^
- Choking is a preventable yet serious emergency; if a foreign object remains in a person’s airway, the person will become hypoxic, and cardiac arrest will follow.^5^
- The Heimlich maneuver may relieve airway obstruction due to a foreign object.^5^

Things to Remember for Seizures

- A seizure consists of abnormal neuronal firing in the brain, resulting in loss of consciousness and usually has a characteristic tonic-clonic muscle activity.^6^
- Ensure the patient is safe during a seizure by laying her on the floor or flat surface, on the side, protecting the airway, and removing potential injury provoking objects.^7^

Things to Remember for Anaphylaxis

- Anaphylaxis is an allergic reaction involving two or more body systems.^8^
- Signs to look for include stridor, inability to phonate, and wheezing.^8^
- To give an epi-pen, remember “blue to the sky and orange to the thigh” and give it through clothing or to the bare skin while holding it for a minimum of 3 seconds.^8^

Things to Remember for Bleeding

- Hemostasis is the process by which the body controls bleeding. The type of bleeding can be arterial, venous, or capillary.^9^
- For most cases of external bleeding, the best immediate step in controlling bleeding is to apply direct pressure, with packing if the wound is deep.^9^
- For extremity bleeding, another strategy may include using a tourniquet.^9^

## Supplementary Information



## Figures and Tables

**Table 1 t1-9-3-sg63:** First Aid Simulation Quiz Results

Question	Pre-curriculum % Correct	Post-curriculum % Correct	p-value
Overall	53.9	82.2	<0.001
Q1	78.9	71.7	0.101
Q2	33.6	80.3	<0.001
Q3	65.8	78.3	0.005
Q4	53.2	92.8	<0.001
Q5	71.1	97.4	<0.001
Q6	38.8	98.0	<0.001
Q7	26.3	41.4	<0.001
Q8	25.7	85.5	<0.001
Q9	74.3	93.4	<0.001
Q10	89.5	99.3	<0.001
Q11	44.1	93.4	<0.001
Q12	66.4	62.5	0.356
Q13	16.4	59.9	<0.001
Q14	69.7	97.4	<0.001

**Table 2 t2-9-3-sg63:** Student Reactions to First Aid Simulation – Self-efficacy and Satisfaction Survey

Statement	Pre-curriculum	Post-curriculum	p-value
I can recognize an airway, breathing, or circulation life threat.	3.39	4.77	<0.001
I can describe at-scene management options for an airway, breathing, or circulation life threat.	2.49	4.72	<0.001
I can describe in-hospital management options for an airway, breathing, or circulation life threat.	2.31	4.43	<0.001
I can provide first aid to a patient experiencing choking.	3.38	4.77	<0.001
I can provide first aid to a patient experiencing a seizure.	2.33	4.81	<0.001
I can provide first aid to a patient hemorrhaging from an extremity.	2.61	4.80	<0.001
I can provide first aid to a patient experiencing anaphylaxis.	2.95	4.83	<0.001

I enjoyed this first aid Workshop.		4.75	
This workshop was a good use of scheduled educational time.		4.79	
The content covered in this workshop is relevant to my future practice.		4.90	
This workshop was an effective way to practice first aid skills.		4.74	
This workshop was an effective way to practice assessing airway, breathing, and circulation.		4.73	
I would recommend this workshop to future medical students.		4.78	

**Figure f1-9-3-sg63:**
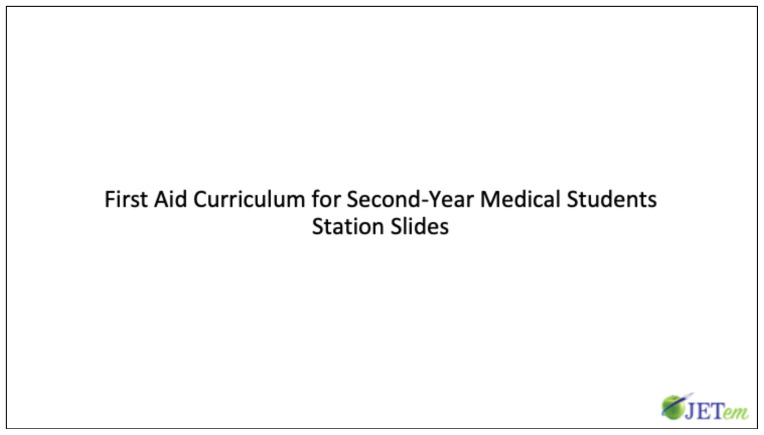
Please see associated PowerPoint file

## SMALL GROUPS LEARNING MATERIALS

### Appendix A Pre-curriculum Questionnaire

**Please provide an identifier consisting of your mother's initials and the last two digits of your phone number (eg, SLL35).** We use this information to match your pre-curriculum and post-curriculum surveys for data analysis, while maintaining your anonymity.

Write your identifier here: ______________________

**Please rate your agreement with the following statements on a scale of 1 to 5**, where 1 = strongly disagree, and 5 = strongly agree.[Table t4-9-3-sg63]

**Table t4-9-3-sg63:**

	Strongly Disagree			Strongly Agree	
I can recognize an airway, breathing, or circulation life threat.	1	2	3	4	5
I can describe at-scene management options for an airway, breathing, or circulation life threat.	1	2	3	4	5
I can describe in-hospital management options for an airway, breathing, or circulation life threat.	1	2	3	4	5
I can provide first aid to a patient experiencing choking.	1	2	3	4	5
I can provide first aid to a patient experiencing a seizure.	1	2	3	4	5
I can provide first aid to a patient hemorrhaging from an extremity.	1	2	3	4	5
I can provide first aid to a patient experiencing anaphylaxis.	1	2	3	4	5

**Please select the most suitable answer for the following multiple-choice questions:**

The goals of first aid are best described as:

- Identifying an airway, breathing, or circulation life threat
- Providing immediate, on-site management for medical emergencies
- Obtaining an accurate history and physical examination
- Observing the patient while waiting for paramedics or another clinical team to arrive

Signs of a threatened airway may include:

- Lack of breath sounds or chest movement
- Wheezing
- Altered breathing pattern
- Inability to speak or phonate

While out on a run, you witness a man collapse to the ground, with occasional myoclonic jerks to his extremities. He is unconscious. What is the best initial action?

- Assess airway, breathing, and circulation
- Check the man’s pockets for identification
- Place the man in the recovery position
- Protect the cervical spine by maintaining alignment

You encounter an assault victim who has sustained a deep laceration to the left arm after being pushed through a glass screen door. He is bleeding profusely from a wound near the left elbow. What is the best initial step to control bleeding?

A. Apply direct pressure
B. Place a tourniquet on the left upper extremity
C. Wrap the arm tightly with compressive dressing
A. Hold his arm upright to decrease blood flow to the arm

Where should you place a tourniquet in relation to a bleeding extremity wound?

- 2 to 3 inches distal to the wound
- Directly on the wound
- 2 to 3 inches proximal to the wound
- 6 to 8 inches proximal to the wound

Which of the following makes the best improvised tourniquet?

- A shoelace
- A belt
- A cloth strip with a windlass
- Multiple rubber bands

While eating out at a restaurant, a woman at the next table chokes on her food. She is coughing loudly and forcefully. What is the next best step?

- Deliver back blows
- Perform the Heimlich maneuver
- Perform a finger sweep if you see food in the mouth
- Monitor the woman

When performing the Heimlich maneuver on a pregnant patient in their third trimester, where should you position your hands?

- On the upper margin of the sternum, near the sternal angle
- On the middle of the sternum, in the center of the sternal body
- On the lower margin of the sternum, near the xiphoid process
- To the left of the sternum, over the likely site of the gastric fundus

Which is the next best step for a choking victim who has lost consciousness and become pulseless?

- Chest compressions
- Finger sweep
- Back blows
- Heimlich maneuver

While you are in a crowded concert, you witness a man fall to the ground with tonic-clonic jerking consistent with a seizure. What is the next best step?

- Put something between his teeth to prevent tongue biting
- Search the man’s pockets for seizure medications
- Ask for help to carry the man out of the event
- Remove nearby objects and people to prevent injury to the man

Which of the following describes the “rescue position”?

- Supine positioning
- Prone positioning
- Left or right lateral recumbent positioning
- Upright in a chair with a pillow behind the head

What is the most common source of airway obstruction in an unconscious patient?

- Spit or other oral secretions
- Vomit or other gastric contents
- An aspirated foreign body
- The tongue

While at a party, your friend accidentally eats a dessert containing peanuts. You know she was hospitalized last year after a severe allergic reaction to peanuts. What is the next best step?

- Administer your friend’s Epi-Pen (epinephrine auto-injector)
- Administer Benadryl (diphenhydramine)
- Try to induce vomiting
- Monitor for signs of an allergic reaction

Which of these following patients is experiencing anaphylaxis?

- A patient reporting abdominal pain and vomiting after eating peanut butter
- A patient reporting shortness of breath after a wasp sting
- A patient with hives and wheezing after being outdoors
- A patient with red, flushed skin after eating shrimp

Where should an epinephrine auto-injector be administered?

- Lateral leg, underneath clothing
- Lateral thigh, through overlying clothing
- Lateral abdomen, underneath clothing
- Left chest, into the heart, through overlying clothing

### Appendix B First Aid Curriculum Station Slides

[Fig f1-9-3-sg63]

### Appendix C Choking/Heimlich Maneuver for Adults, Children, and Infants

#### Activity Summary

In this 20-minute small group activity, a facilitator will lead a group of five to six medical students through a clinical scenario featuring a choking patient. Students are expected to perform an airway, breathing, circulation assessment and interactively work with the facilitator to manage the patient, including performing the Heimlich maneuver.

#### Educational Objectives

By the end of this activity, learners will be able to:

- Describe airway, breathing, and circulation findings suggestive of foreign body aspiration
- Describe the universal sign for choking and other clinical signs of choking
- Demonstrate the Heimlich maneuver in an adult
- Describe adjustments to the Heimlich maneuver in special patient populations, specifically patients who are pregnant or obese
- Demonstrate back slaps and chest thrusts in an infant

#### Equipment Needs

- Adult low-fidelity manikin (eg, low-fidelity CPR manikin)
- Infant low-fidelity manikin
- Monitor /laptop for visual cues

#### Facilitation Guide

##### Station Timing

[Table t5-9-3-sg63]

**Table t5-9-3-sg63:**

Time	Activity
2–3 minutes	Introduction
5 minutes	A/B/C Assessment
5 minutes	Recognizing the Emergency
5 minutes	Choking First Aid
2–3 minutes	Wrap-Up / Summary

##### Introduction

The facilitator should introduce themselves to the group and describe the goals of the station (learning how to provide first aid to a choking person), and then provide a clinical case to frame the discussion. A suggested script might be:

*You’re at a Christmas party. There are all sorts of fun, themed foods, including eggnog, gingerbread cookies, and Grinch fruit kabobs: a green grape, banana slice, and strawberry on a toothpick topped with a mini marshmallow. As you’re talking with a person who is enjoying the fruit kabob, they become silent all the sudden, their face turns red, and they cross their hands in front of their neck.*

Facilitator can then show the first image as a visual aid: Go to choking section in First Aid Curriculum Slides.

##### A/B/C Assessment

The facilitator should lead the group through an airway, breathing, and circulation assessment of the patient: Ask, “What do you think you would observe regarding the person’s airway, breathing, and circulation for someone in this situation?”

- Airway: The patient is unable to make any noise, including no choking and no coughing noises.
- Breathing: You see that they have some small movements of the chest wall, but you don’t see any significant rise and fall of the chest. You don’t see any cyanosis of the lips or face.
- Circulation: The patient looks to have warm skin and intact peripheral pulses.

Clinical implications:

- Airway: Complete airway obstruction
- Breathing: Compromised due to complete airway obstruction, but likely no hypoxia yet. Cyanosis is a late finding and indicates impending decline.
- Circulation: Intact, but could become compromised with prolonged airway obstruction.

##### Recognizing the Emergency

The facilitator should lead the group through the following key discussion questions:

1. Ask the students, “How can you tell if this is an emergency?”
  - Look for “universal choking sign” (hands crossed over throat)
  - Ask patient to speak - inability to speak or otherwise phonate is strongly suggestive of a complete airway obstruction
  - Other signs of airway obstruction may include: stridor, weak cough, or cry
2. Ask the students, “What could happen with an unresolved, complete airway obstruction?”
  - Complete airway obstruction would eventually result in hypoxia, ending in cardiac arrest
  - Incomplete airway obstruction could still result in aspiration, leading to pneumonia, pneumonitis, and other respiratory sequelae
3. Ask the students, “If the patient was still coughing or making some noise, would that change your airway assessment?”
  - May be consistent with an incomplete airway obstruction
  - If cough is weak and the patient cannot speak or cry, should treat as a complete airway obstruction
  - If coughing strongly, appropriate treatment may be monitoring and calling for help
4. Ask the students, “What can you do to help this patient?”
  - Heimlich maneuver
  - Back blow

##### Choking First Aid

The facilitator should guide learners through the Heimlich maneuver in adults and children, including time to practice the maneuver.

1. Ask the students, “What is the Heimlich maneuver?”
  - Use choking section in First Aid Curriculum Slides as a visual aid
  - Goal is to use abdominal thrusts to elevate the diaphragm and increase airway pressure, forcing air from the lungs
  - If successful, this forces an artificial cough, expelling a foreign body from the airway
2. Ask the students, “How is the Heimlich maneuver performed in adults and children >1 year of age?”
  - Heimlich steps:
    - Make fist with one hand
    - Place thumb side of the fist against the patient’s abdomen, in the midline above the navel and below the xiphoid process (essential to prevent unnecessary internal injury)
    - Grasp fist with other hand
    - Pull fist into the patient’s abdomen with a quick inward and upward thrust
    - Repeat until either the object is expelled or the victim becomes unresponsive
  - Have students practice maneuver on each other, or on the low-fidelity adult manikin if they prefer
3. Ask the students what they would do next if the patient loses consciousness.
  - American Heart Association guidelines recommend the following sequence of steps
  - Active emergency response system
  - Then:
    - Attempt ventilation
    - Perform Heimlich maneuver up to five times - use choking section in First Aid Curriculum Slides as a visual aid
    - Perform tongue-jaw lift and finger sweep
    - Repeat above until obstruction is cleared, advanced medical help arrives, or the patient loses pulses
    - Have the students practice these maneuvers on the minikin. The jaw thrust can be practiced with a peer if they choose.
4. Ask the students what would they do next if the patient loses pulses.
  - American Heart Association guidelines recommend the following sequence of steps
  - Active emergency response system
  - Then:
  - Perform chest compressions and attempt ventilations at 30:2 ratio
    - After each attempt at ventilation, perform tongue-jaw lift and finger sweep
    - Repeat above until obstruction is cleared or advanced medical help arrives
  - Of note, CPR probably generates similar intrathoracic pressure as the Heimlich maneuver, and it may also be effective in dislodging a foreign body in the airway; it may be reasonable to just start CPR if the patient becomes unconscious
5. Ask the students, “Is there ever a time when the standard positioning of a Heimlich maneuver would not be appropriate in a choking person? What about pregnant or obese patients?”
  - Use choking section in First Aid Curriculum Slides as a visual aid
  - Pregnancy elevates diaphragm, and the girth of the gravid uterus may prevent rescuer from circling the abdomen; similarly, it may be difficult to encircle the abdomen if the patient is obese
  - The fist should go in the center of the sternum, avoiding the xiphoid process and the margins of the rib cage
6. What about infants <1 year of age?
  - Use choking section in First Aid Curriculum Slides as a visual aid
  - Alternate between five back blows and five chest thrusts
  - Have students practice on the infant manikin

Discussion points that may come up:

- Should back slaps be performed? Unclear - American Heart Association vs. European Resuscitation Council guidelines differ. Some say to alternate between five thrusts of the Heimlich maneuver with five back slaps.
- If the patient loses consciousness:
  - Should finger sweeps be performed? American Heart Association guidelines state yes, if the provider is clinically trained /not a layperson.
  - Should the Heimlich maneuver still be performed? American Heart Association guidelines state yes, if 1) the provider is clinically trained /not a layperson, and 2) the patient still has a pulse

##### Wrap-Up / Summary

At the conclusion of the activity, wrap up with the following points:

- Signs of choking include the universal signal for choking, inability to speak, coughing, labored breathing, and changes in coloration.
- Choking is a preventable yet serious emergency; if a foreign object remains in a person’s airway, they will become hypoxic, and cardiac arrest will follow.
- The Heimlich maneuver may relieve airway obstruction due to a foreign object:
  - In adults and children >1 year old, position your hands between the umbilicus and the xiphoid process.
  - In pregnant women or obese patients, position your hands over the mid-sterum.
  - In infants <1 year old, alternate between back blows and chest thrusts.

#### Potential Facilitation Challenges

- Timing of the session may be an issue. If there is not enough time, facilitators should prioritize discussing the signs of choking and how to perform the Heimlich maneuver. If there is free time at the end of the session, facilitators can discuss reasons why a person may be at increased risk for choking.
- Focus on asking students questions and having them practice as opposed to reading the guide to them. Fill in their knowledge gaps with the information provided in this guide.

#### References

- https://www.ahajournals.org/doi/full/10.1161/circ.102.suppl_1.I-22
- https://www.ahajournals.org/doi/10.1161/circ.102.suppl_1.I-253#d3e2867

### Appendix D Seizure/Recovery Position

#### Activity Summary

In this 20-minute small group activity, a facilitator will lead a group of five to six medical students through a clinical scenario featuring a patient having a seizure. Students are expected to perform an airway, breathing, circulation assessment and interactively work with the facilitator to manage the patient, including placing the patient in the appropriate recovery position.

#### Educational Objectives

By the end of this activity, learners will be able to:

- Describe airway, breathing, and circulation findings suggestive of tongue obstruction
- Describe clinical features of a generalized, tonic-clonic seizure
- Implement strategies to assist an individual experiencing seizure, specifically timing the seizure, removing nearby dangerous objects, placing the patient in the recovery position, and protecting the head and airway

#### Equipment Needs

- Stopwatch
- Low-fidelity manikin - positioned with tables/chairs nearby as potentially harmful objects
- Cushion/pillow/sweatshirt to place under patient’s head
- Monitor /laptop for playing video and showing slides

#### Facilitation Guide

##### Station Timing

[Table t6-9-3-sg63]

**Table t6-9-3-sg63:**

Time	Activity
2–3 minutes	Introduction
5 minutes	A/B/C Assessment
3 minutes	Recognizing the Emergency
7 minutes	Seizure First Aid
2–3 minutes	Wrap-Up / Summary

##### Introduction

The facilitator should introduce themselves to the group and describe the goals of the station (learning how to provide first aid to an individual experiencing a generalized, tonic-clonic seizure), and then provide a clinical case to frame the discussion. A suggested script might be:

*You are in class and about to take a scheduled NBME examination. As you get settled, you notice the student next to you stiffen and suddenly begin convulsing, with rapid jerking movements of their face, eyelids, arms, and legs.*

Facilitator can then show the following video as a visual aid: Go to seizure section in First Aid Curriculum Slides.

##### A/B/C Assessment

The facilitator should lead the group through an airway, breathing, and circulation assessment of the patient: Ask, “What do you think you would observe regarding the person’s airway, breathing, and circulation for someone in this situation?”

- Airway: You notice some frothy and blood-tinged spit coming from your classmate’s mouth. He’s occasionally vocalizing but it sounds like grunting. He does not meaningfully respond with speech.
- Breathing: Your classmate seems to be breathing spontaneously.
- Circulation: Your classmate has warm skin and intact peripheral pulses.

Clinical implications:

- Airway: Potentially has an issue, as the patient is currently unable to handle secretions. However, vocalizations are a positive sign.
- Breathing: Intact
- Circulation: Intact

##### Recognizing the Emergency

The facilitator should lead the group through the following key discussion questions:

1. Ask the students, “Is this an emergency? How can you tell?”
  - Generalized tonic-clonic seizures reflect uncontrolled electrical activity in the brain. Findings may include:
    - Loss of consciousness
    - Convulsions of all extremities, with tonic (flexion/extension of extremities, tremor) and clonic (contraction and relaxation of muscles) phases
    - Facial twitching /jaw clenching
    - Tongue laceration
    - Post-ictal period
  - If generalized seizure occurs without interruption (status epilepticus), patients can end up with brain injury or death
  - Seizure activity may lead to airway obstruction, apneic pauses, and increased oxygen requirements, which could culminate in critical hypoxia

##### Seizure First Aid

The facilitator should guide learners through some initial interventions that can be provided to actively seizing patients:

1. Ask the students, “What can you do to help someone who is actively seizing?” Inform them of any points they did not come up with on their own.
  - Call for help
  - Time the seizure
  - Remove nearby obstacles
  - Cushion and protect the head
  - Reassess airway, breathing, and circulation
  - Stay with the individual
  - Place in recovery position, if possible
  - Do not restrain the patient nor attempt to remove foam from the mouth during a seizure because it can cause aspiration.
2. Ask the students, “What is the expected course of a seizure? What does the post-ictal period look like clinically?”
  - The majority of seizures spontaneously terminate within 5 minutes.
  - Patients are expected to have a post-ictal state with decreased consciousness and confusion afterwards, lasting a variable amount of time, but usually no more than minutes.
3. Ask the students, “What can you do to help someone who is post-ictal?”
  - Reassess airway, breathing, and circulation
  - Reassure the individual
  - Place in recovery position - seizure section in First Aid Curriculum Slides
  - Have students practice getting into recovery position - seizure section in First Aid Curriculum Slides
4. Ask students, “What is the most common source of airway obstruction in an unconscious or obtunded person?”
  - The tongue
  - The recovery position can help relieve airway obstruction by:
    - Lifting the tongue from the back of the throat
    - Preventing saliva or aspirated gastric contents from going down the airway

##### Wrap-Up / Summary

At the conclusion of the activity, wrap up with the following points:

- A generalized tonic-clonic seizure consists of abnormal neuronal firing in the brain, resulting in loss of consciousness and characteristic tonic / clonic muscle activity.
- Ensure the patient is safe during a seizure by laying her on the floor/flat surface, on the side, protecting the airway, and removing potential injury provoking objects.

#### Potential Facilitation Challenges

- Lack of time: Prioritize emphasizing the treatment measures.
- Too much time: Have students practice placing each other in the rescue position and become familiar with the rescue medication. Quiz students on the wrap-up points.
- Focus on asking students questions and having them practice as opposed to reading the guide to them. Fill in their knowledge gaps with the information provided in this guide.

#### Media and Resources for Facilitators

- https://my.clevelandclinic.org/health/diseases/22788-tonic-clonic-grand-mal-seizure
- https://www.epilepsy.com/treatment/seizure-rescue-therapies

#### References

- Bank AM, Bazil CW. Emergency management of epilepsy and seizures. *Semin Neurol*. 2019 Feb;39(1):73–81. At: doi: 10.1055/s-0038-1677008. Epub 2019 Feb 11. PMID: 30743294.
- Falco-Walter J. Epilepsy-definition, classification, pathophysiology, and epidemiology. *Semin Neurol*. 2020 Dec;40(6):617–623. Epub 2020 Nov 5. PMID: 33155183. At: doi: 10.1055/s-0040-1718719

### Appendix E Anaphylaxis/Epinephrine Pen Injections

#### Activity Summary

In this 20-minute small group activity, a facilitator will lead a group of five to six medical students through a clinical scenario featuring a patient experiencing anaphylaxis. Students are expected to perform an airway, breathing, circulation assessment and interactively work with the facilitator to manage the patient, including epipen administration.

#### Educational Objectives

By the end of this activity, learners will be able to:

- Define anaphylaxis as an allergic reaction involving two or more organ systems
- Identify signs of airway compromise, including tachypnea, wheezing, and stridor
- Demonstrate safe and appropriate use of an epinephrine auto-injector

#### Equipment Needs

- Medium- to high-fidelity manikin with breath sound
- Practice epi-pen injector
- Monitor /laptop for playing video and showing slides

#### Facilitation Guide

##### Station Timing

[Table t7-9-3-sg63]

**Table t7-9-3-sg63:**

Time	Activity
2–3 minutes	Introduction
5 minutes	A/B/C Assessment
5 minutes	Recognizing the Emergency
5 minutes	Anaphylaxis First Aid
2–3 minutes	Wrap-Up / Summary

##### Introduction

The facilitator should introduce themselves to the group and describe the goals of the station (learning how to provide first aid to an individual experiencing anaphylaxis), and then provide a clinical case to frame the discussion. A suggested script might be:

*You are working as a summer camp instructor as the campers are playing Capture the Flag. One of the campers sprints across the field with the other team’s flag – they win! As other campers celebrate, the one holding the flag can’t seem to catch his breath, and eventually crumples over. You rush over to see that his skin is developing a rash, and he is making a high-pitched noise with each rapid breath. His pulse is rapid and weak.*

Facilitator can then show the following image/video as a visual aid: Go to anaphylaxis section in First Aid Curriculum Slides

##### A/B/C Assessment

The facilitator should lead the group through an airway, breathing, and circulation assessment of the patient: Ask, “What do you think you would observe regarding the person’s airway, breathing, and circulation for someone in this situation?”

- Airway: The camper is making a high-pitched noise with each breath and seems to have trouble speaking. He looks distressed and anxious.
- Breathing: The camper’s breathing is rapid.
- Circulation: You feel warm skin and intact peripheral pulses.

Clinical implications:

- Airway: Ask students, “What is the clinical term for the high-pitched noise with each breath?” The noise is consistent with stridor, which is a sign of airway obstruction. The patient’s inability to speak is a bad sign. In combination, the symptoms point to impending airway obstruction.
- Breathing: Compromised due to airway obstruction, but intact for now.
- Circulation: Intact but could be compromised with prolonged airway obstruction.

##### Recognizing the Emergency

The facilitator should lead the group through the following key discussion questions:

1. Ask the students, “Is this an emergency? How can you tell?”
  - Yes, patient is showing clear signs of airway compromise, which must be immediately addressed.
2. Ask the students, “What could be going on with the patient?”
  - Anaphylaxis - a severe type 1 hypersensitivity reaction mediated by mast cell degranulation and histamine release. It is a reaction that involves two or more body systems.
  - Ask the students, “What findings would you expect to see in the different organ systems?” Prompt them with the specific organ systems listed below if needed.
    - Cardiac: tachycardia, hemodynamic collapse
    - Respiratory: bronchospasm + wheezing
    - Gastrointestinal: nausea + vomiting
    - Skin: urticaria, flushing, angioedema
    - Neurologic: confusion (likely due to shock)
3. Ask the students, “What are some of the ways anaphylaxis leads to life threats?” Prompt them with the ABCs if needed.
  - Airway: Discuss warning signs of upper airway edema, ie, stridor with inspiration vs expiration, mucosal edema
  - Breathing: Discuss anaphylaxis-related bronchospasm and herald signs for respiratory collapse, ie, respiratory rate, inspiratory vs expiratory wheezing, accessory muscle use
  - Circulation: Discuss signs of distributive shock, ie, pallor, tachycardia, weak pulse, syncope, altered mentation

##### Anaphylaxis First Aid

The facilitator should guide learners through management of anaphylaxis with timely administration of epinephrine, including practice with a simulated epinephrine auto-injector.

1. Ask the students, “How is anaphylaxis managed?”
  - Immediate administration of epinephrine
    - First and most important treatment
    - Should be administered immediately upon recognition of anaphylaxis
  - Adjuncts include: H1 blockers, H2 blockers, steroids, IV fluids, inhaled bronchodilators
2. Ask the students, “How can we deliver epinephrine in a first aid setting?”
  - A variety of commercial epinephrine auto-injectors exist, such as Epi-Pens
  - Discuss use of an Epi-Pen:
    - Correct identification of the safety cap (blue) and the injector (orange)
    - Avoidance of placing thumbs/fingers over ends - manufacturer states this is to prevent accidentally injecting yourself if you mix up the safety cap end vs. the injector end in the heat of the moment
    - Removal of blue safety cap
    - Selection of lateral middle thigh site and delivery through clothing
    - Importance of holding firmly against the thigh for 3 seconds following needle deployment
3. Ask the students, “What does the patient need after epinephrine injection?”
  - Emergency medicine services for transport to a safe monitoring site, such as a hospital
  - Possibility of recurrent episodes requiring repeat dosage of epinephrine-auto injector

##### Wrap-Up /Summary

At the conclusion of the activity, wrap up with the following points:

- Anaphylaxis is an allergic reaction involving two or more organ systems
- Signs of airway compromise may include stridor and inability to phonate
- Signs of respiratory/breathing compromise may include wheezing and tachypnea
- Review steps of safely administering an Epi-Pen:
  - “Blue to the sky, orange to the thigh”
  - Avoid covering the ends of the auto-injector to avoid self-injury
  - Administer through clothing
  - Hold firmly against thigh for a minimum of 3 seconds

#### Potential Facilitation Challenges

- Highlighting distinction between mild allergic symptoms and potentially life-threatening allergies /anaphylaxis– ie, when to intervene versus when to watch.
- Distinguishing between stridor and wheezing and identifying relevant skin findings.
- Focus on asking students questions and having them practice as opposed to reading the guide to them. Fill in their knowledge gaps with the information provided in this guide.

#### Media and Resources for Facilitators

- Airway Management
  - https://www.ncbi.nlm.nih.gov/books/NBK470403/
  - https://accessemergencymedicine.mhmedical.com/content.aspx?bookid=683&sectionid=45343644
- Skin images (Urticaria)
  - https://acaai.org/allergies/allergic-conditions/skin-allergy/hives/
- Epi-Pen Insert:
  - https://www.lovell.fhcc.va.gov/MedicalHomeport/PediatricDocs/EpipenInstructions.pdf

#### References

- https://www.ncbi.nlm.nih.gov/books/NBK470403/
- https://accessemergencymedicine.mhmedical.com/content.aspx?bookid=683&sectionid=45343644
- https://acaai.org/allergies/allergic-conditions/skin-allergy/hives/
- https://www.lovell.fhcc.va.gov/MedicalHomeport/PediatricDocs/EpipenInstructions.pdf

### Appendix F Bleeding/Pressure, Packing, Tourniquet

#### Activity Summary

In this 20-minute small group activity, a facilitator will lead a small group consisting of five to six medical students through a clinical scenario of a patient presenting with life-threatening external hemorrhage. Students will be expected to perform an airway, breathing, circulation assessment as the facilitator teaches the students the clinical pearls of how to manage bleeding. The facilitator will also demonstrate how to stop bleeding with basic interventions, such as direct pressure, packing, and tourniquet application.

#### Educational Objectives

By the end of the activity, learners will be able to

- Describe airway, breathing, and circulation findings suggestive of external hemorrhage
- Describe indications of hemorrhagic shock
- Demonstrate readily accessible interventions for controlling external hemorrhage, such as direct pressure and packing
- Demonstrate tourniquet placement with a commercial and improvised tourniquet

#### Equipment Needs

- Gauze for demonstrating wound packing
- Commercial tourniquet
- Improvised tourniquet supplies: tie or similar wide band of cloth, wooden spoon

#### Facilitation Guide

##### Station Timing

[Table t8-9-3-sg63]

**Table t8-9-3-sg63:**

Time	Activity
2–3 minutes	Introduction
5 minutes	A/B/C Discussion
2–3 minutes	Recognizing the Emergency
7 minutes	Bleeding First Aid
2–3 minutes	Wrap-Up/Summary

##### Introduction

The facilitator should introduce themselves to the group and describe the goals of the station (learning how to provide first aid to an individual experiencing life-threatening bleeding), and then provide a clinical case to frame the discussion. A suggested script might be:

*sAs you are cheering on the finishers for a marathon, you hear a loud explosion, and a cloud of dust covers the finish line. Chaos ensues as bystanders scream and begin to flee the scene. You notice a man calling for help who has a large wound to his right arm, with exposed bone and loss of tissue. He is bleeding profusely, with dark blood gushing out of the wound.*

##### A/B/C Assessment

The facilitator should lead the group through an airway, breathing, and circulation assessment of the patient: Ask, “What do you think you would observe regarding the person’s airway, breathing, and circulation for someone in this situation?”

- Airway: The patient is speaking to you in complete sentences.
- Breathing: The patient has symmetric chest rise and a normal respiratory rate.
- Circulation: The patient looks increasingly pale, and he is exhibiting some confusion, with delayed responses to questions.

Clinical implications:

- Airway: Intact
- Breathing: Intact
- Circulation: Beginning to exhibit signs of shock, such as pallor and altered mental status. Other findings may include tachycardia, delayed capillary refill, and cool extremities.

##### Recognizing the Emergency

The facilitator should lead the group through the following key discussion questions:

1. Ask the students, “Is this an emergency? How can you tell?”
  - Uncontrolled hemorrhage will lead to loss of circulating volume, followed by shock, followed by death
  - Hemostasis is defined as the arrest of bleeding and is the primary goal when dealing with a patient with a major source of bleeding
2. Ask the students, “What are potential types of bleeding that this patient may be experiencing?” Prompt them to think about the color of the blood based on its current location in the body if needed
  - Venous blood will be more oozing and will be dark
  - Arterial blood will be spurting and bright red
  - Capillary blood may be a trickle, have initially fast blood flow, but will be easily controlled
  - Of note, it is possible that this patient has internal bleeding; however, as a first aid provider, you can only address external bleeding and attempt to transport the patient to a higher level of care.

##### Bleeding First Aid

The facilitator should guide learners through initial interventions that can be provided for external sources of hemorrhage.

1. Ask the students, “For most situations, what is the best initial step for controlling external hemorrhage?”
  - Direct pressure on the wound using cloth, gauze, or saline soaked sponge
  - Must apply more pressure than the pressure of the venous, arterial, or capillary bleeding in order to promote clot formation and hemostasis
  - For deep wounds, packing with direct pressure may be required to achieve hemostasis - Go to bleeding section in First Aid Curriculum Slides for a visual aid
  - Periodically re-evaluate the status of the bleeding by lessening pressure
  - Have students practice with each other with gauze
2. Ask students, “What are other initial interventions for controlling hemorrhage from an extremity?”
  - Raising the extremity above the heart can help promote blood flow back to heart using gravity and improve perfusion of critical organs
  - If bleeding continues, consider use of a tourniquet
3. Ask the students, “How can you apply a commercial tourniquet?”
  - Discuss parts of a commercial tourniquet
    - Can use bleeding section in First Aid Curriculum Slides for a visual aid
4. Ask the students, “What if you do not have a commercial tourniquet available?”
  - Improvised tourniquets are a reasonable option, though they must be applied correctly to have reliability
  - MUST have a windlass (such as a wooden spoon or stick) to effectively increase pressure above arterial pressure
  - Can use bleeding section in First Aid Curriculum Slides for a visual aid
  - Set aside time for practice between students with both the commercial tourniquet and improvised tourniquet

##### Wrap-Up /Summary

At the conclusion of the activity, wrap up with the following points:

- Hemostasis is the process by which the body controls bleeding. The type of bleeding can be arterial, venous, or capillary
- For most cases of external bleeding, the best immediate step in controlling bleeding is to apply direct pressure, with packing if the wound is deep
- For extremity bleeding, other strategies may include raising the extremity and using a tourniquet

#### Potential Challenges

- Facilitators may have trouble engaging students through the A/B/C assessment in relation to the case because of the lack of clinical background of the first year medical students. Facilitators need to be aware of this and help keep the activity moving forward in the event students are not answering questions appropriately within the given time frame.
- Facilitators may have trouble with time when demonstrating how to stop bleeding. For this reason, facilitators should try and leave 5–10 minutes to allow students to.demonstrate what they’ve learned, including stopping bleeding with gauze, applying the tourniquet and creating a makeshift tourniquet.
- With the group consisting of six medical students, the facilitator may face the challenge of making sure each student feels involved. The case is based on interactive learning, so the facilitator needs to ensure each group member is involved throughout the activity.
- Focus on asking students questions and having them practice as opposed to reading the guide to them. Fill in their knowledge gaps with the information provided in this guide.

#### Media and Resources for Facilitators

- How to place a tourniquet:https://www.crisis-medicine.com/improvised-tourniquets-good-to-have-an-alternate-plan/

#### References

- Donley ER, Loyd JW. Hemorrhage Control. [Updated 2021 Jul 23]. In: StatPearls [Internet]. Treasure Island (FL): StatPearls Publishing; 2022 Jan. Available from: https://www.ncbi.nlm.nih.gov/books/NBK535393/
- Johnson AB, Burns B. Hemorrhage. [Updated 2022 May 4]. In: StatPearls [Internet]. Treasure Island (FL): StatPearls Publishing; 2022 Jan. Available from: https://www.ncbi.nlm.nih.gov/books/NBK542273/
- Resources Poster Booklet. Stop The Bleed. Available from: https://www.stopthebleed.org/resources-poster-booklet/
- LaPelusa A, Dave HD. Physiology, hemostasis. [Updated 2022 May 8]. In: StatPearls [Internet]. Treasure Island (FL): StatPearls Publishing; 2022 Jan. Available from: https://www.ncbi.nlm.nih.gov/books/NBK545263/

### Appendix G Post-Curriculum Questionnaire

**Please provide an identifier consisting of your mother's initials and the last two digits of your phone number (eg, SLL35).** We use this information to match your pre-curriculum and post-curriculum surveys for data analysis, while maintaining your anonymity.

Write your identifier here: ______________________

**Please rate your agreement with the following statements on a scale of 1 to 5**, where 1 = strongly disagree, and 5 = strongly agree.[Table t9-9-3-sg63]

**Table t9-9-3-sg63:**

	Strongly Disagree			Strongly Agree	
I can recognize an airway, breathing, or circulation life threat.	1	2	3	4	5
I can describe at-scene management options for an airway, breathing, or circulation life threat.	1	2	3	4	5
I can describe in-hospital management options for an airway, breathing, or circulation life threat.	1	2	3	4	5
I can provide first aid to a patient experiencing choking.	1	2	3	4	5
I can provide first aid to a patient experiencing a seizure.	1	2	3	4	5
I can provide first aid to a patient hemorrhaging from an extremity.	1	2	3	4	5
I can provide first aid to a patient experiencing anaphylaxis.	1	2	3	4	5

**Please select the most suitable answer for the following multiple-choice questions:**

The goals of first aid are best described as:

- Identifying an airway, breathing, or circulation life threat
- Providing immediate, on-site management for medical emergencies
- Obtaining an accurate history and physical examination
- Observing the patient while waiting for paramedics or another clinical team to arrive

Signs of a threatened airway may include:

- Lack of breath sounds or chest movement
- Wheezing
- Altered breathing pattern
- Inability to speak or phonate

While out on a run, you witness a man collapse to the ground, with occasional myoclonic jerks to his extremities. He is unconscious. What is the best initial action?

- Assess airway, breathing, and circulation
- Check the man’s pockets for identification
- Place the man in the recovery position
- Protect the cervical spine by maintaining alignment

You encounter an assault victim who has sustained a deep laceration to the left arm after being pushed through a glass screen door. He is bleeding profusely from a wound near the left elbow. What is the best initial step to control bleeding?

- Apply direct pressure
- Place a tourniquet on the left upper extremity
- Wrap the arm tightly with compressive dressing
- Hold his arm upright to decrease blood flow to the arm

Where should you place a tourniquet in relation to a bleeding extremity wound?

- 2 to 3 inches distal to the wound
- Directly on the wound
- 2 to 3 inches proximal to the wound
- 6 to 8 inches proximal to the wound

Which of the following makes the best improvised tourniquet?

- A shoelace
- A belt
- A cloth strip with a windlass
- Multiple rubber bands

While eating out at a restaurant, a woman at the next table chokes on her food. She is coughing loudly and forcefully. What is the next best step?

- Deliver back blows
- Perform the Heimlich maneuver
- Perform a finger sweep if you see food in the mouth
- Monitor the woman

When performing the Heimlich maneuver on a pregnant patient in their third trimester, where should you position your hands?

- On the upper margin of the sternum, near the sternal angle
- On the middle of the sternum, in the center of the sternal body
- On the lower margin of the sternum, near the xiphoid process
- To the left of the sternum, over the likely site of the gastric fundus

Which is the next best step for a choking victim who has lost consciousness and become pulseless?

- Chest compressions
- Finger sweep
- Back blows
- Heimlich maneuver

While you are in a crowded concert, you witness a man fall to the ground with tonic-clonic jerking consistent with a seizure. What is the next best step?

- Put something between his teeth to prevent tongue biting
- Search the man’s pockets for seizure medications
- Ask for help to carry the man out of the event
- Remove nearby objects and people to prevent injury to the man

Which of the following describes the “rescue position”?

- Supine positioning
- Prone positioning
- Left or right lateral recumbent positioning
- Upright in a chair with a pillow behind the head

What is the most common source of airway obstruction in an unconscious patient?

- Spit or other oral secretions
- Vomit or other gastric contents
- An aspirated foreign body
- The tongue

While at a party, your friend accidentally eats a dessert containing peanuts. You know she was hospitalized last year after a severe allergic reaction to peanuts. What is the next best step?

- Administer your friend’s Epi-Pen (epinephrine auto-injector)
- Administer Benadryl (diphenhydramine)
- Try to induce vomiting
- Monitor for signs of an allergic reaction

Which of these following patients is experiencing anaphylaxis?

- A patient reporting abdominal pain and vomiting after eating peanut butter
- A patient reporting shortness of breath after a wasp sting
- A patient with hives and wheezing after being outdoors
- A patient with red, flushed skin after eating shrimp

Where should an epinephrine auto-injector be administered?

- Lateral leg, underneath clothing
- Lateral thigh, through overlying clothing
- Lateral abdomen, underneath clothing
- Left chest, into the heart, through overlying clothing

**Please rate your agreement with the following statements on a scale of 1 to 5**, where 1 = strong disagreement, and 5 = strong agreement:[Table t10-9-3-sg63]

**Table t10-9-3-sg63:**

	Strongly Disagree			Strongly Agree	
I enjoyed this First Aid Workshop.	1	2	3	4	5
This workshop was a good use of scheduled educational time.	1	2	3	4	5
The content covered in this workshop is relevant to my future practice.	1	2	3	4	5
This workshop was an effective way to practice first aid skills.	1	2	3	4	5
This workshop was an effective way to practice assessing airway, breathing, and circulation.	1	2	3	4	5
I would recommend this workshop to future medical students.	1	2	3	4	5

**Please give us any additional positive or constructive feedback on the course:**

### Appendix H Multiple Choice Answer Key

**Please select the single best option for the following multiple-choice questions:**

Q1. The goals of first aid are best described as:

- Identifying an airway, breathing, or circulation life threat
- **Providing immediate, on-site management for medical emergencies**
- Obtaining an accurate history and physical examination
- Observing the patient while waiting for paramedics or another clinical team to arrive

Q2. Signs of a threatened airway may include:

- Lack of breath sounds or chest movement
- Wheezing
- Altered breathing pattern
- **Inability to speak or phonate**

Q3. While out on a run, you witness a man collapse to the ground, with occasional myoclonic jerks to his extremities. He is unconscious. What is the best initial action?

- **Assess airway, breathing, and circulation**
- Check the man’s pockets for identification
- Place the man in the recovery position
- Protect the cervical spine by maintaining alignment

Q4. You encounter an assault victim who has sustained a deep laceration to the left arm after being pushed through a glass screen door. He is bleeding profusely from a wound near the left elbow. What is the best initial step to control bleeding?

A. **Apply direct pressure**
B. Place a tourniquet on the left upper extremity
C. Wrap the arm tightly with compressive dressing
A. Hold his arm upright to decrease blood flow to the arm

Q5. Where should you place a tourniquet in relation to a bleeding extremity wound?

- 2 to 3 inches distal to the wound
- Directly on the wound
- **2 to 3 inches proximal to the wound**
- 6 to 8 inches proximal to the wound

Q6. Which of the following makes the best improvised tourniquet?

- A shoelace
- A belt
- **A cloth strip with a windlass**
- Multiple rubber bands

Q7. While eating out at a restaurant, a woman at the next table chokes on her food. She is coughing loudly and forcefully. What is the next best step?

- Deliver back blows
- Perform the Heimlich maneuver
- Perform a finger sweep if you see food in the mouth
- **Monitor the woman**

Q8. When performing the Heimlich maneuver on a pregnant patient in their third trimester, where should you position your hands?

- On the upper margin of the sternum, near the sternal angle
- **On the middle of the sternum, in the center of the sternal body**
- On the lower margin of the sternum, near the xiphoid process
- To the left of the sternum, over the likely site of the gastric fundus

Q9. Which is the next best step for a choking victim who has lost consciousness and become pulseless?

- **Chest compressions**
- Finger sweep
- Back blows
- Heimlich maneuver

Q10. While you are in a crowded concert, you witness a man fall to the ground with tonic-clonic jerking consistent with a seizure. What is the next best step?

- Put something between his teeth to prevent tongue biting
- Search the man’s pockets for seizure medications
- Ask for help to carry the man out of the event
- **Remove nearby objects and people to prevent injury to the man**

Q11. Which of the following describes the “rescue position”?

- Supine positioning
- Prone positioning
- **Left or right lateral recumbent positioning**
- Upright in a chair with a pillow behind the head

Q12. What is the most common source of airway obstruction in an unconscious patient?

- Spit or other oral secretions
- Vomit or other gastric contents
- An aspirated foreign body
- **The tongue**

Q13. While at a party, your friend accidentally eats a dessert containing peanuts. You know she was hospitalized last year after a severe allergic reaction to peanuts. What is the next best step?

- **Administer your friend’s Epi-Pen (epinephrine auto-injector)**
- Administer Benadryl (diphenhydramine)
- Try to induce vomiting
- Watch for signs of an allergic reaction

Q14. Which of these following patients is experiencing anaphylaxis?

- A patient reporting abdominal pain and vomiting after eating peanut butter
- A patient reporting shortness of breath after a wasp sting
- **A patient with hives and wheezing after being outdoors**
- A patient with red, flushed skin after eating shrimp

Q.15 Where should an epinephrine auto-injector be administered?

- Lateral leg, underneath clothing
- **Lateral thigh, through overlying clothing**
- Lateral abdomen, underneath clothing
- Left chest, into the heart, through overlying clothing
