# Supplementary material for: First Aid Curriculum for Second Year Medical Students
Source: J Educ Teach Emerg Med. 2024 Jul 31;9(3):SG63–SG104. doi: 10.21980/J8FH2J (PMC11312873; doi:10.21980/J8FH2J)

## Slide 1
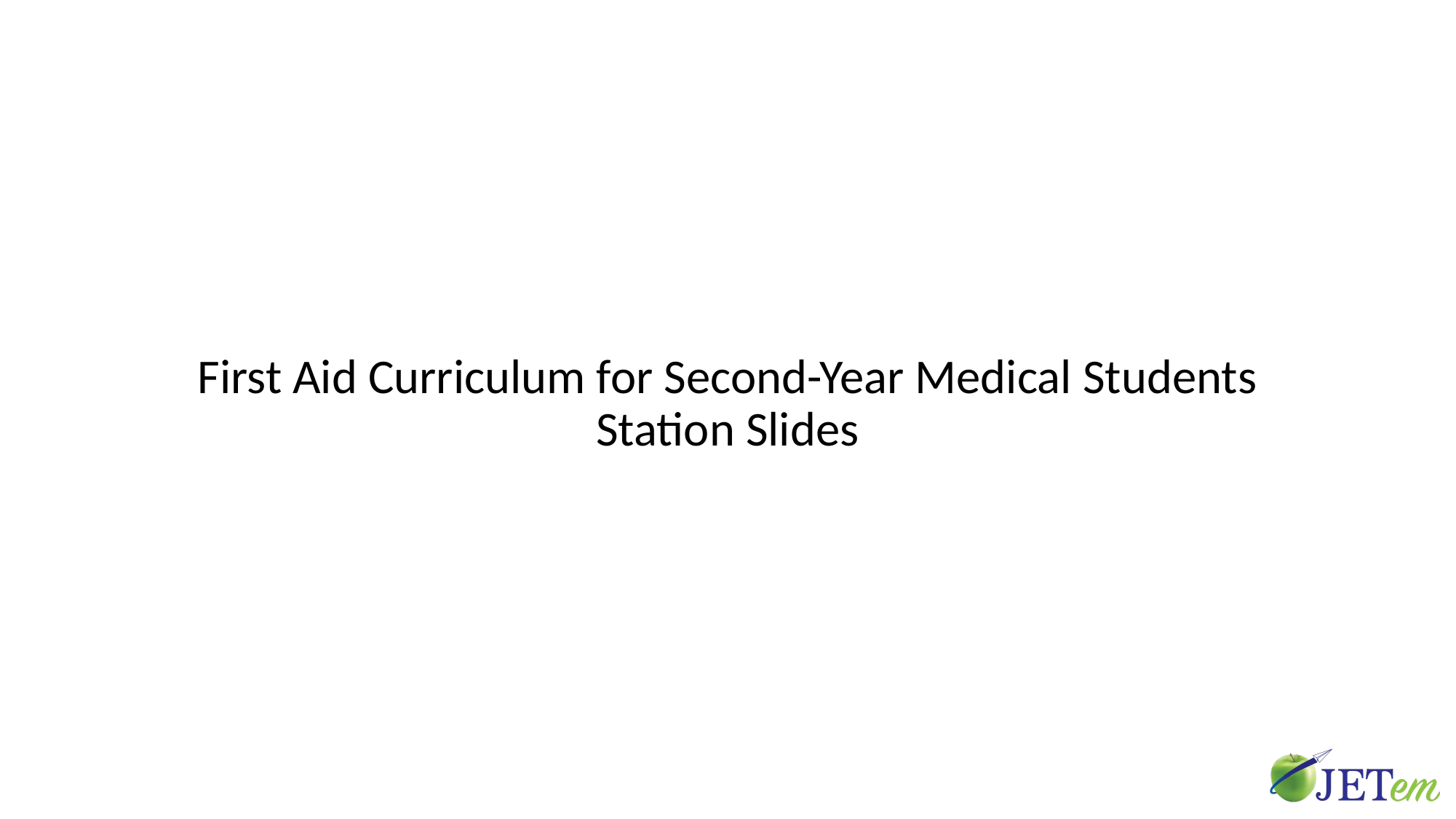

# First Aid Curriculum for Second-Year Medical Students Station Slides

## Slide 2
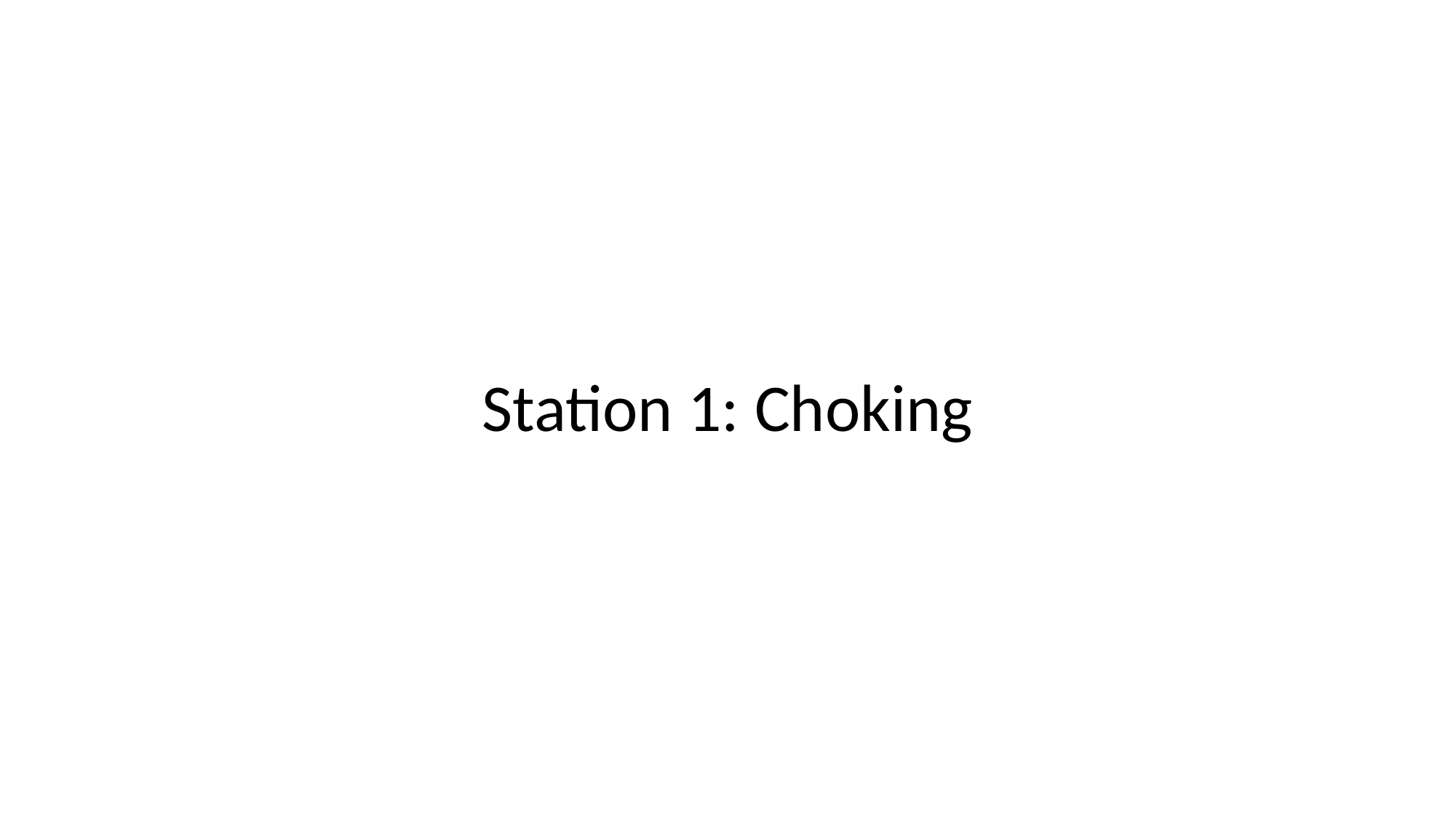

# Station 1: Choking

## Slide 3
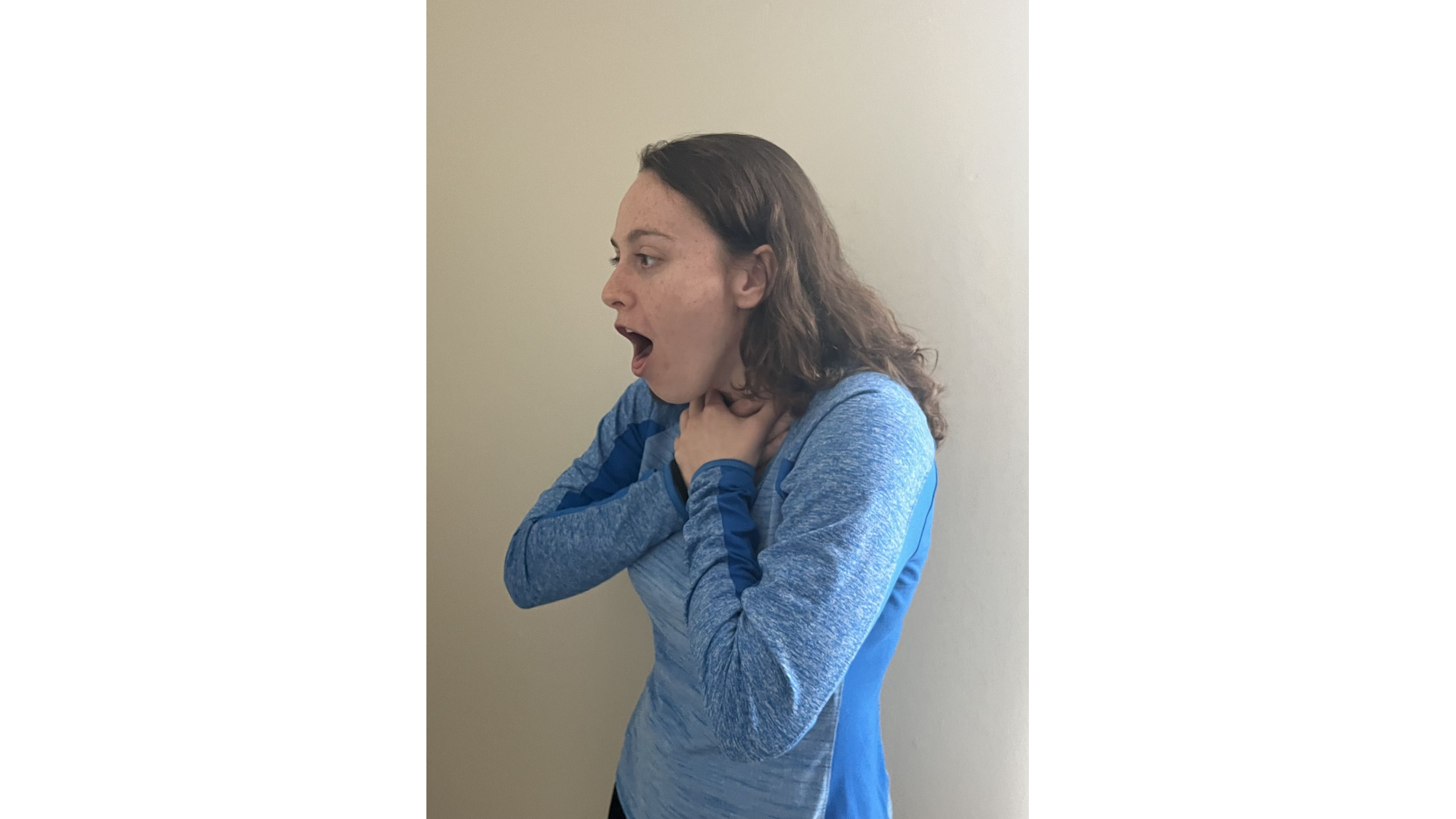

## Slide 4
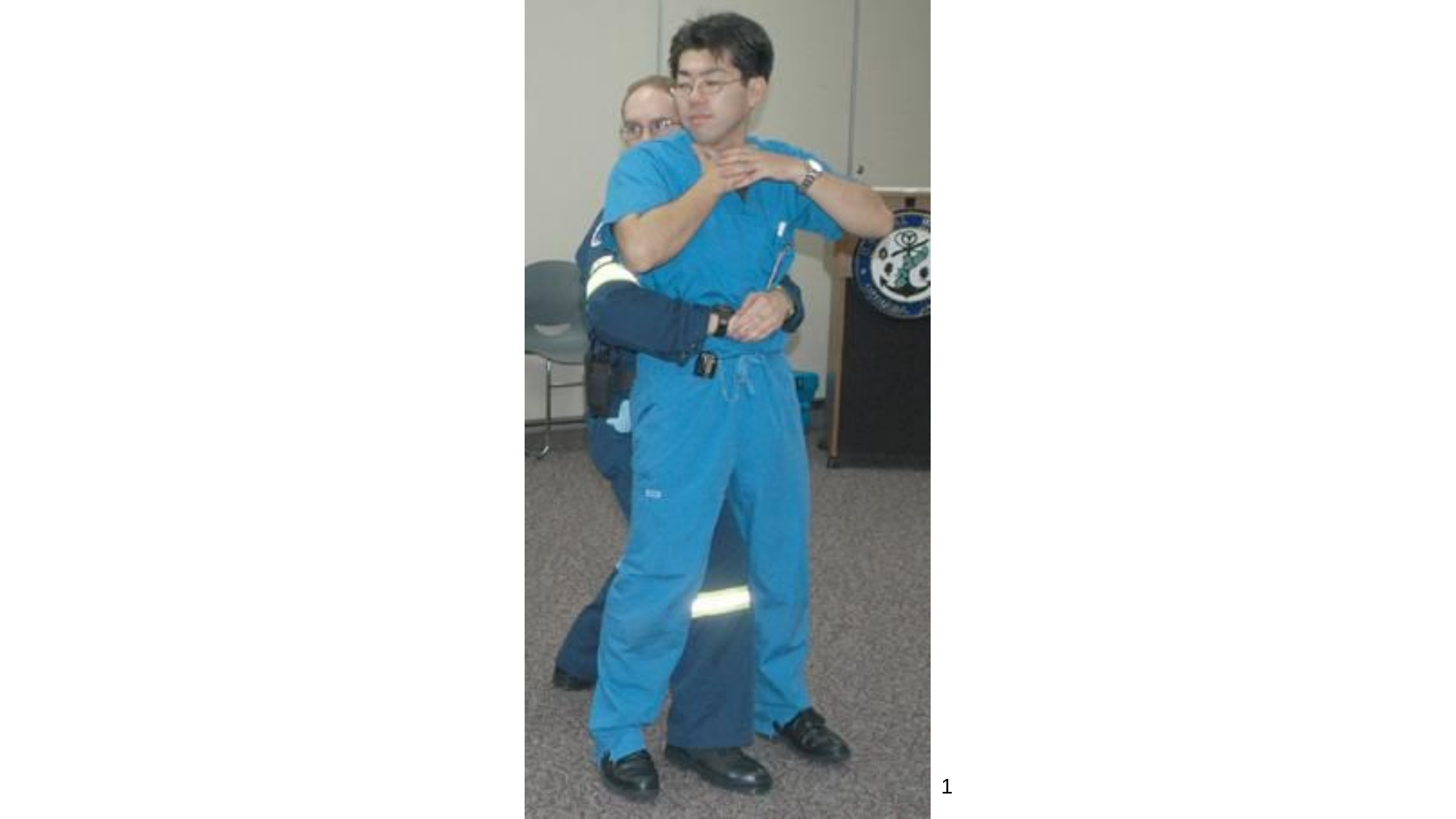

1

## Slide 5
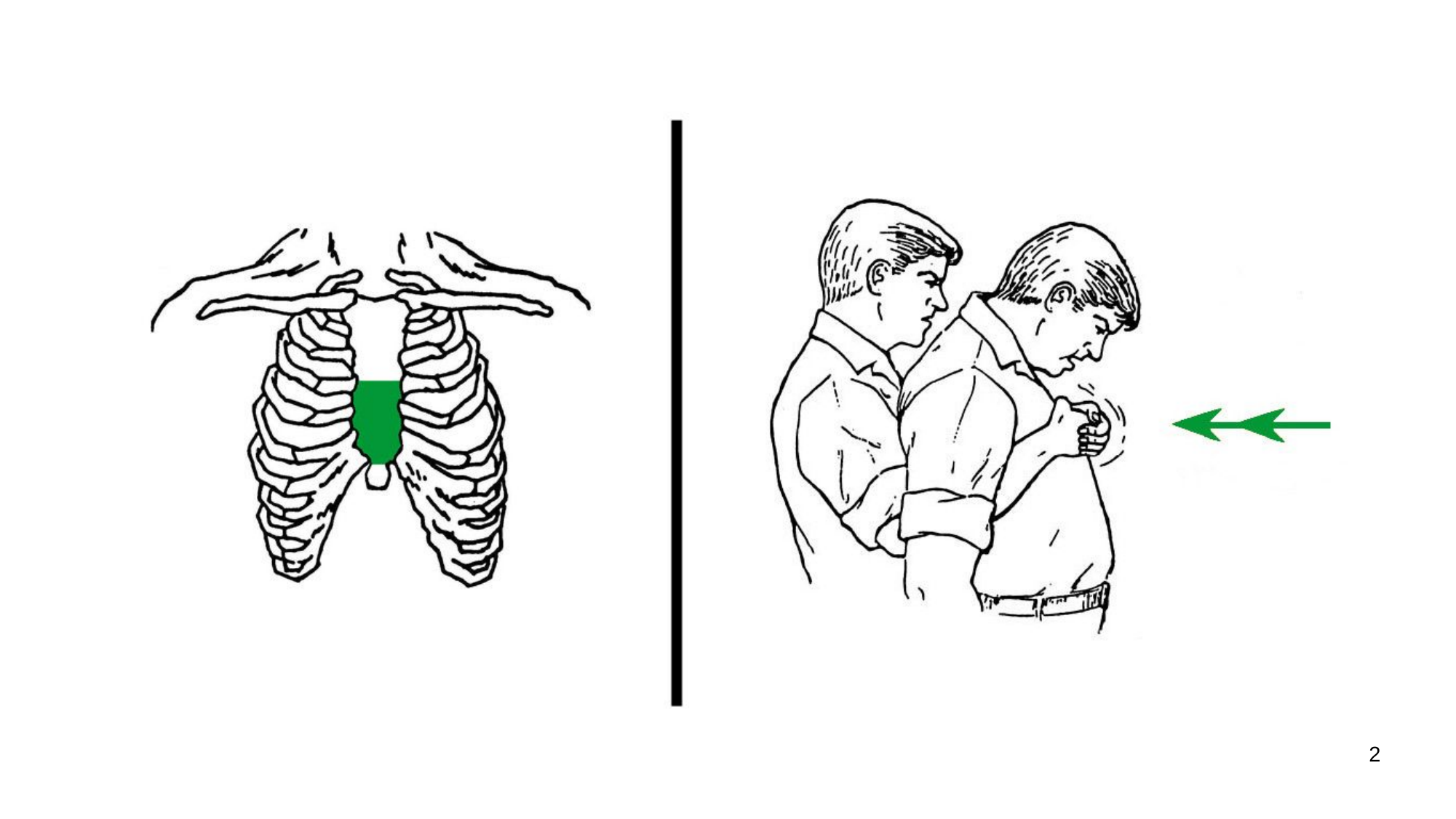

2

## Slide 6
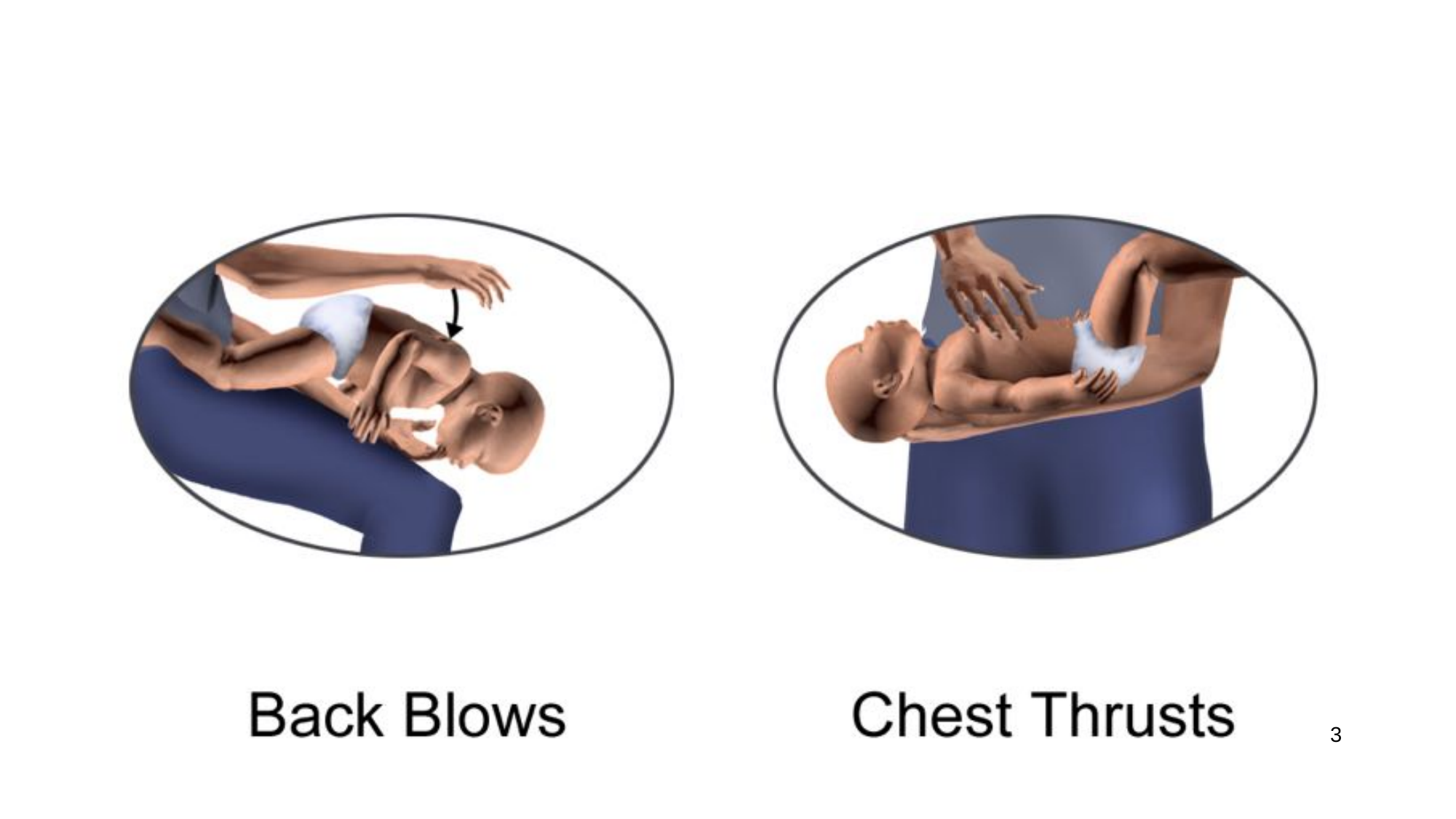

3

## Slide 7
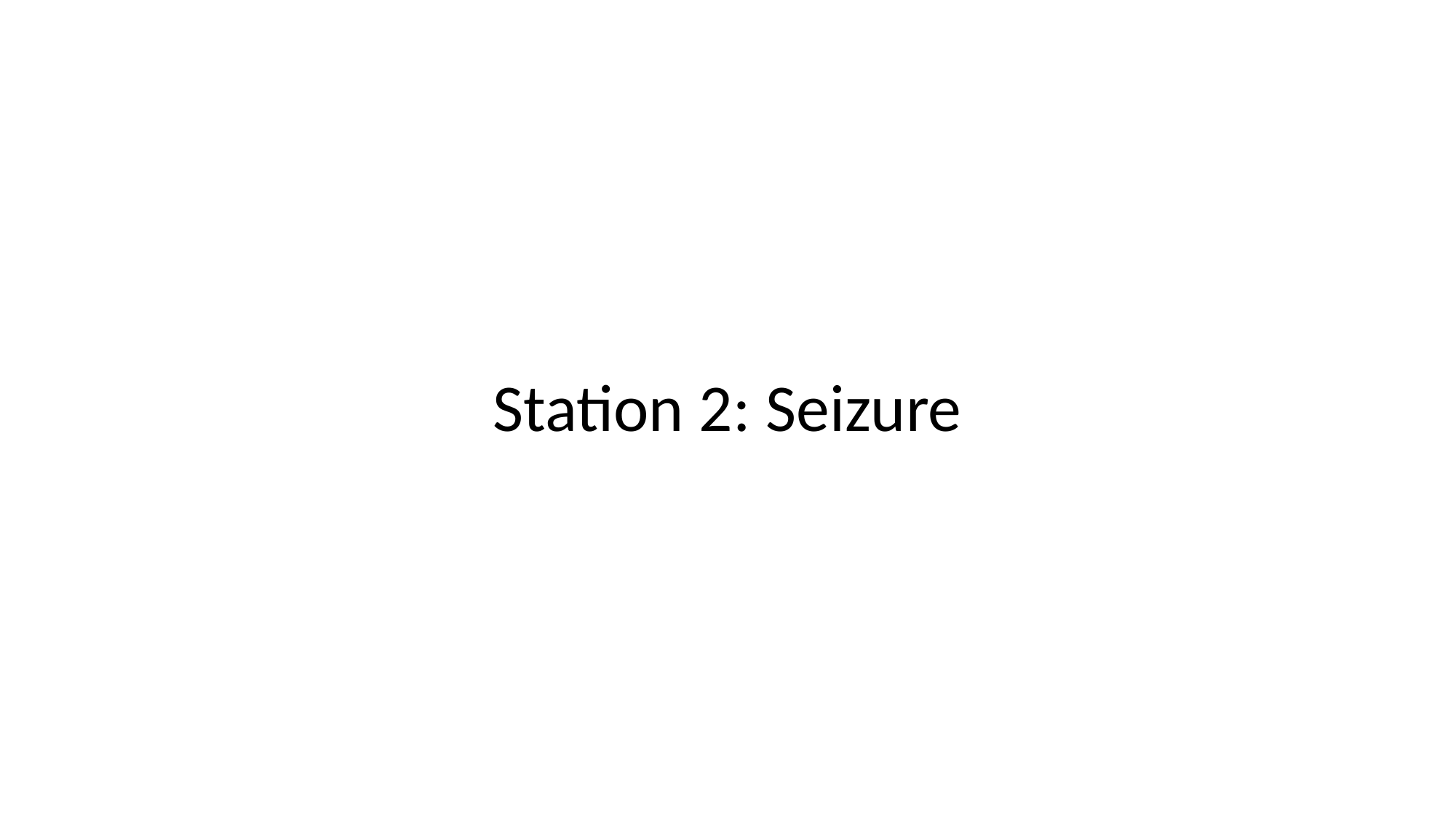

# Station 2: Seizure

## Slide 8
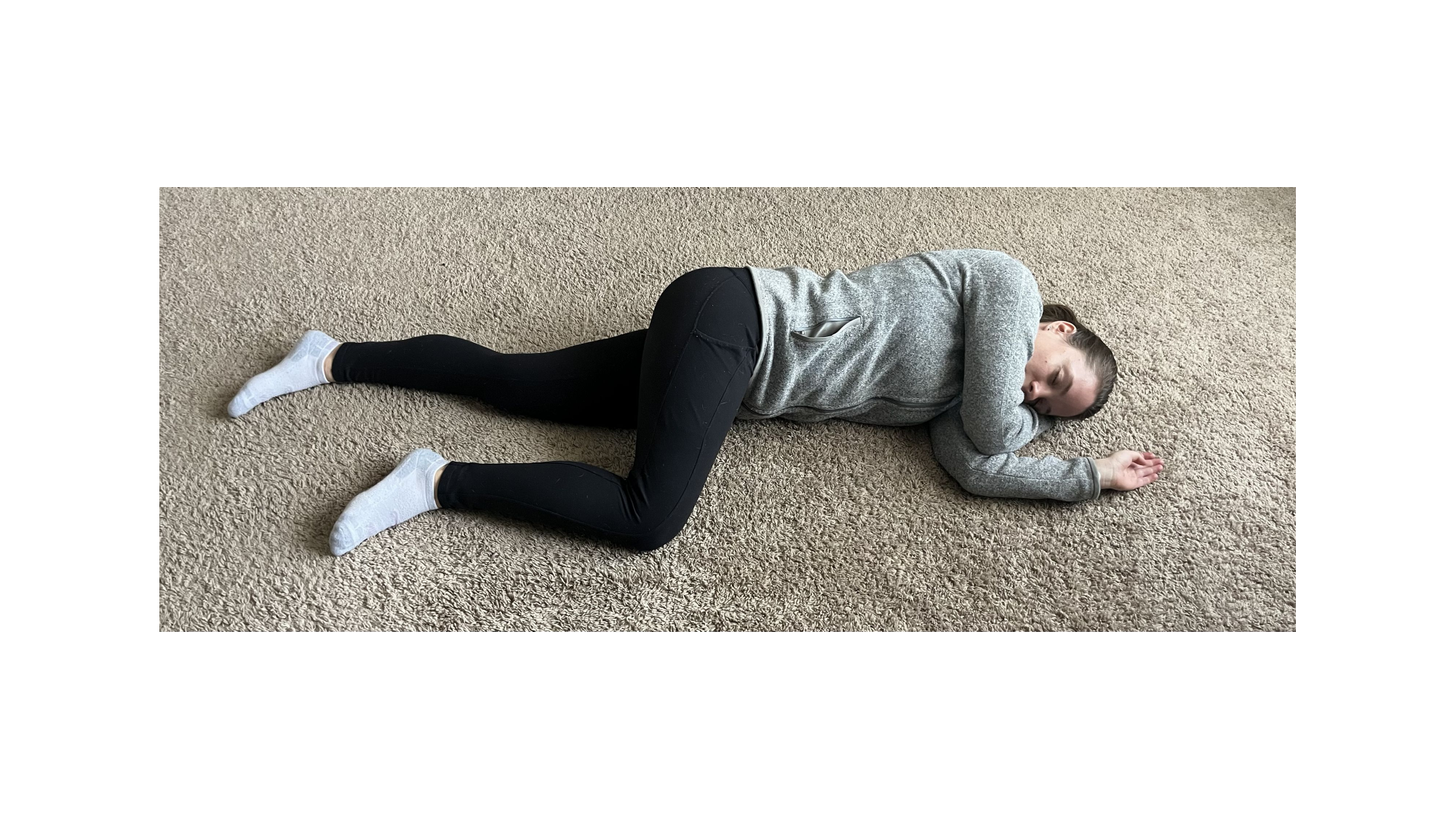

## Slide 9
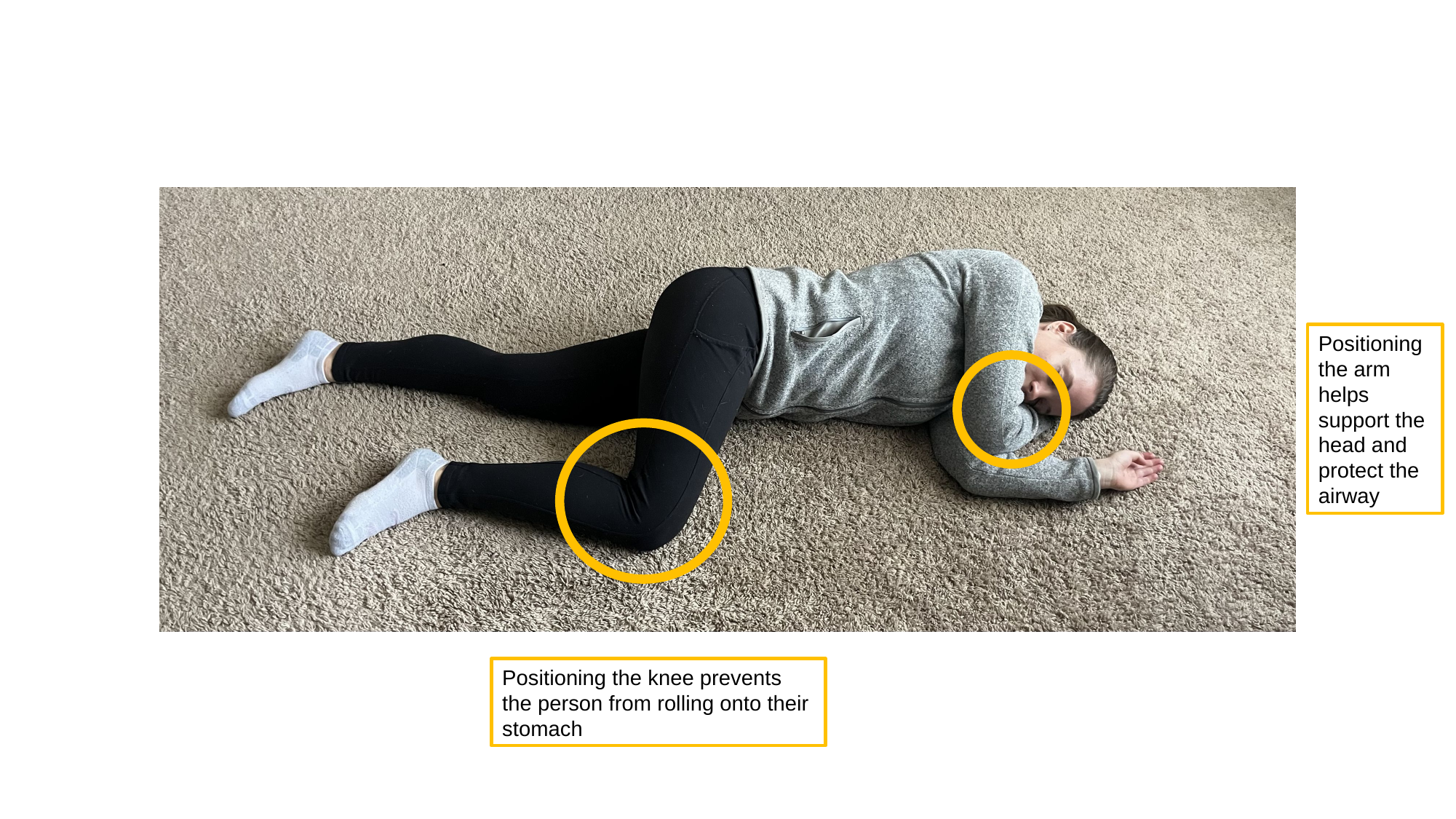

Positioning the arm helps support the head and protect the airway
Positioning the knee prevents the person from rolling onto their stomach

## Slide 10
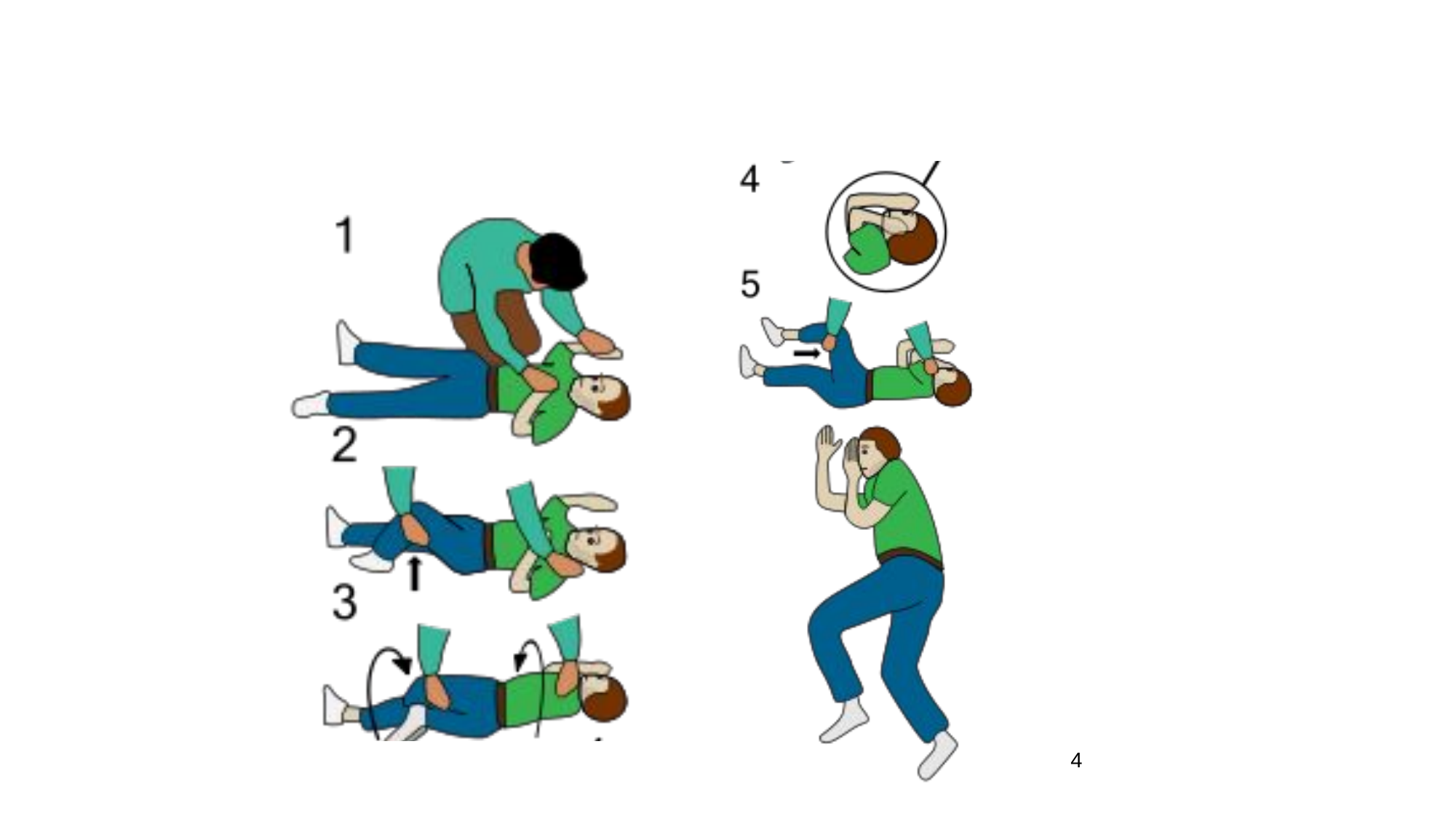

4

## Slide 11
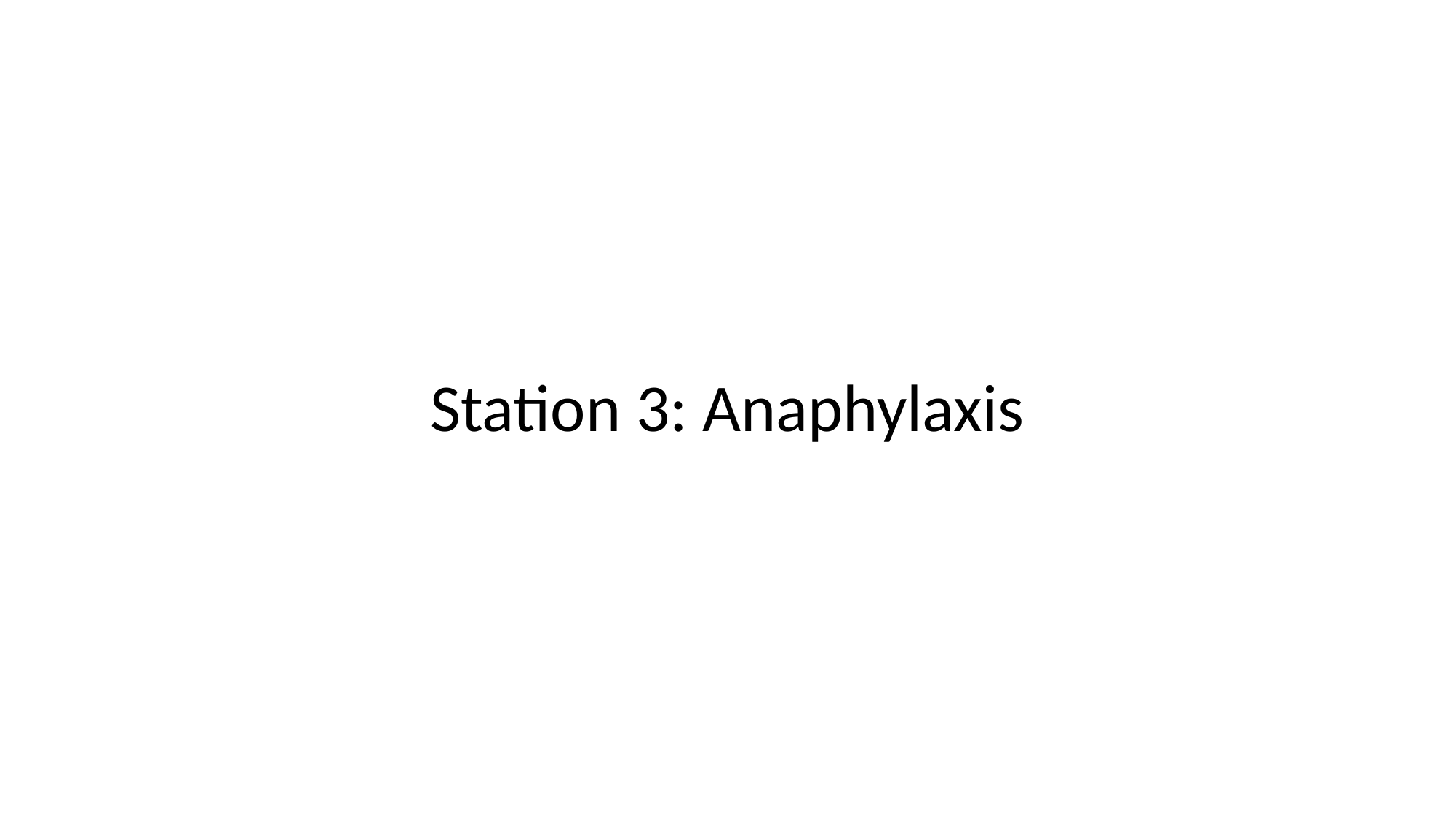

# Station 3: Anaphylaxis

## Slide 12
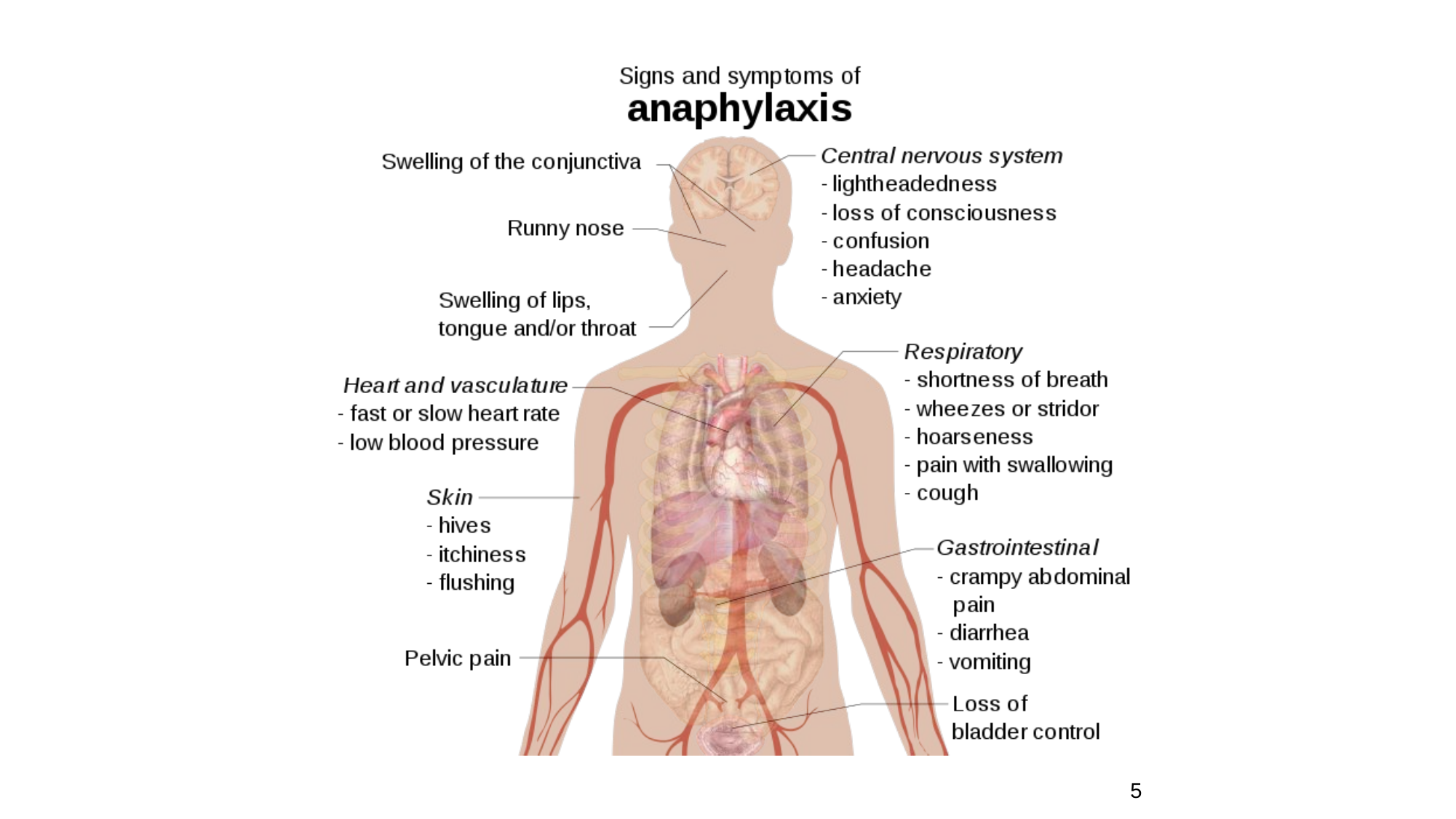

5

## Slide 13
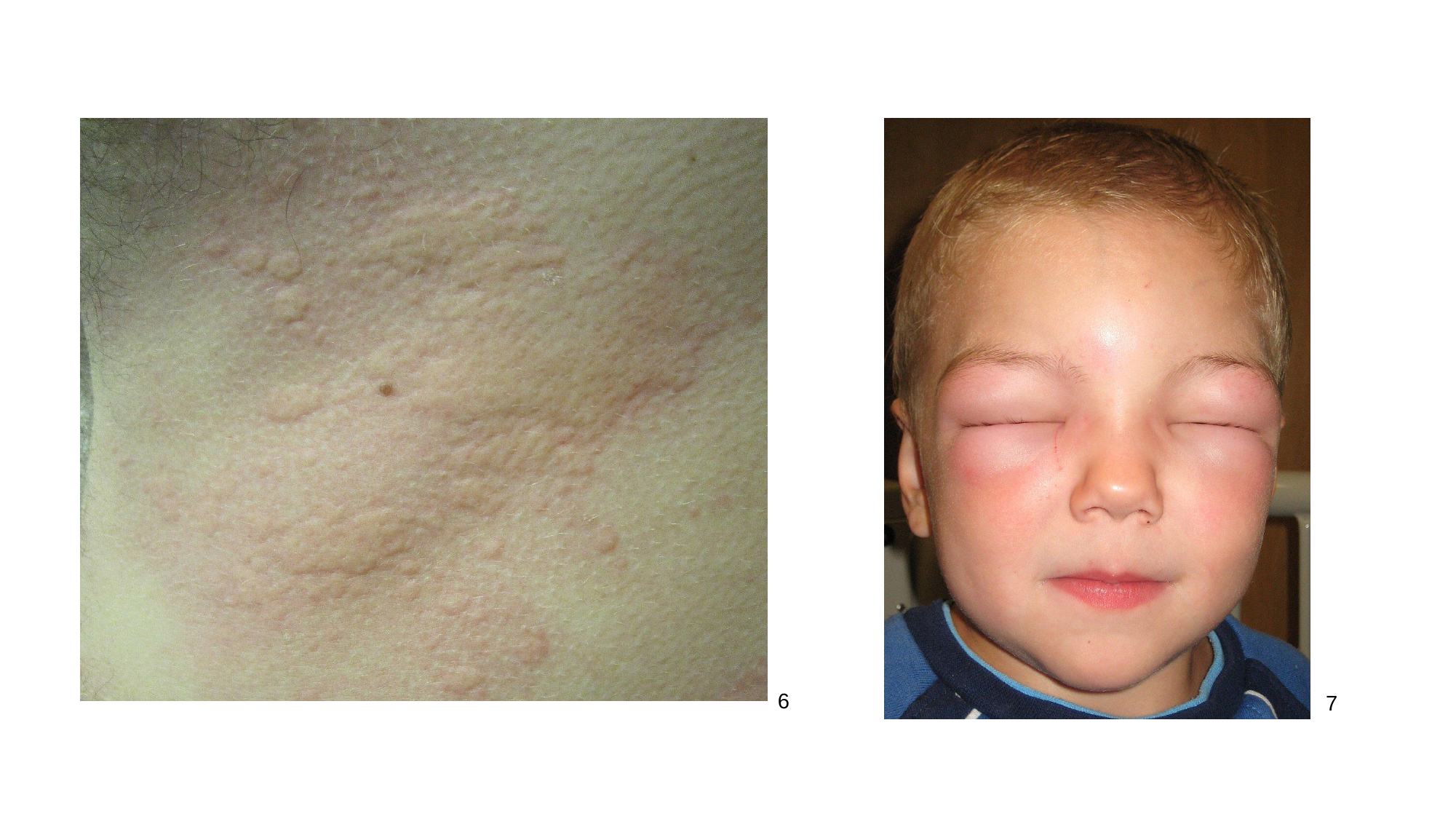

6
7

## Slide 14
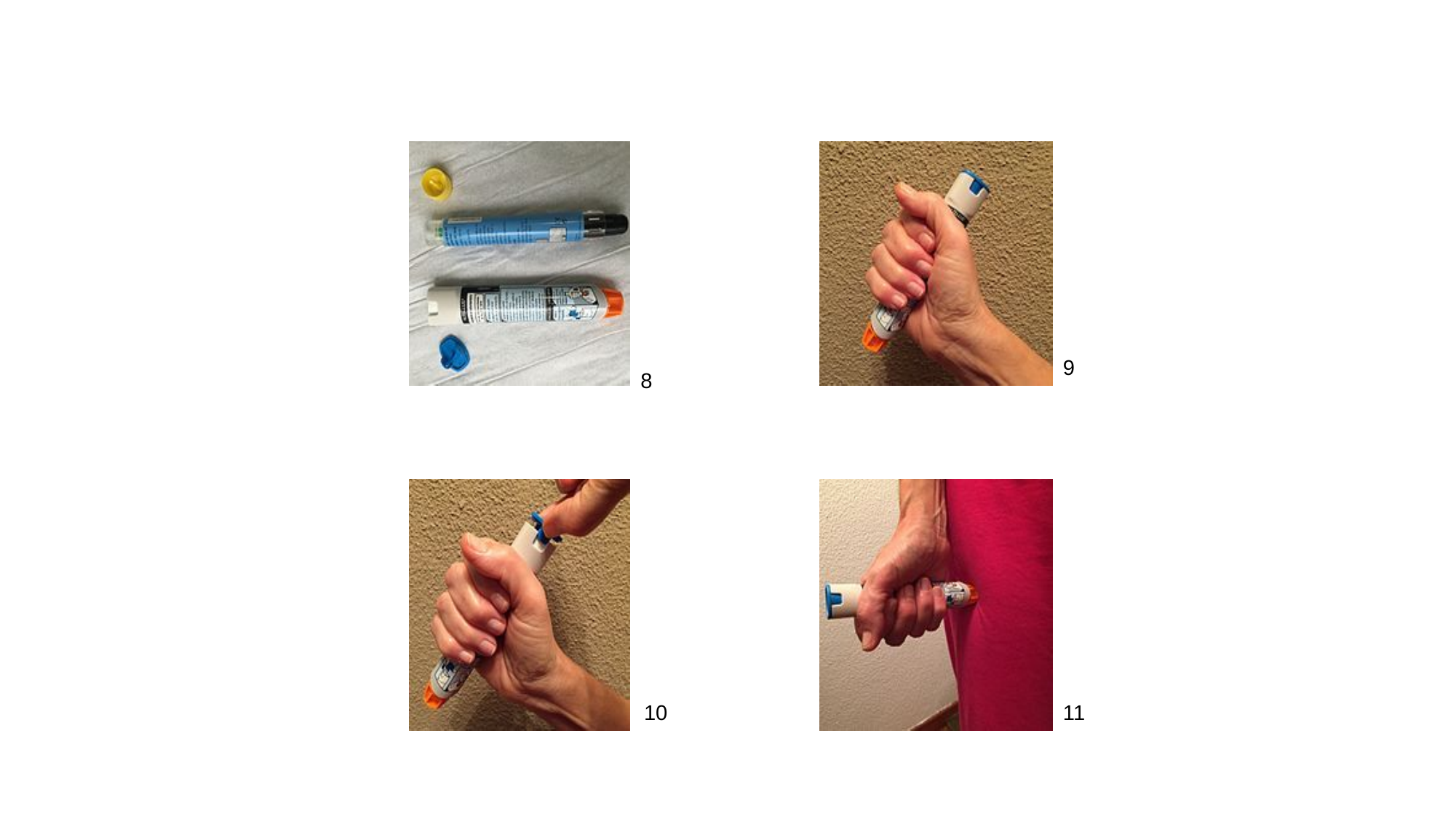

9
8
10
11

## Slide 15
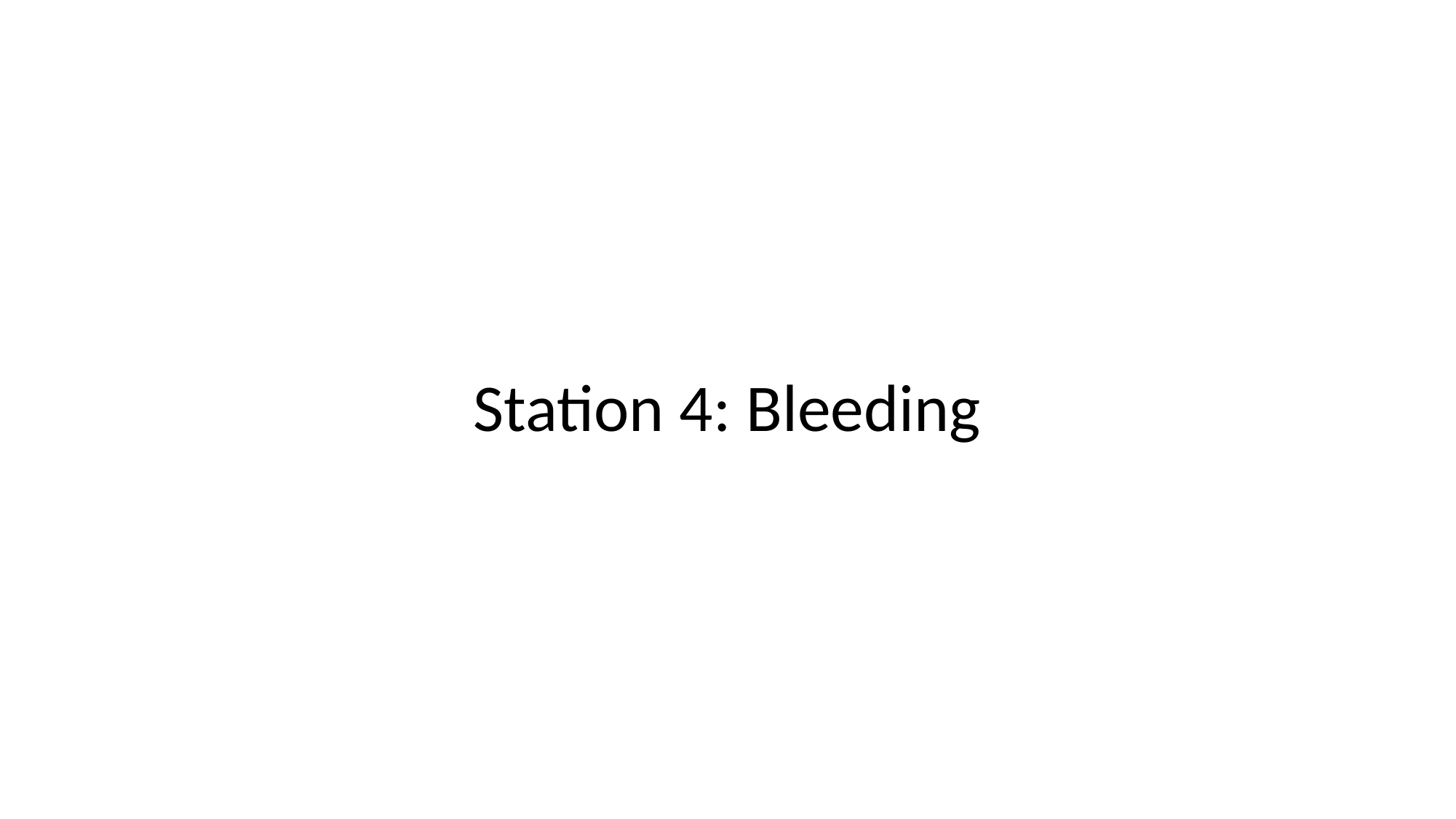

# Station 4: Bleeding

## Slide 16
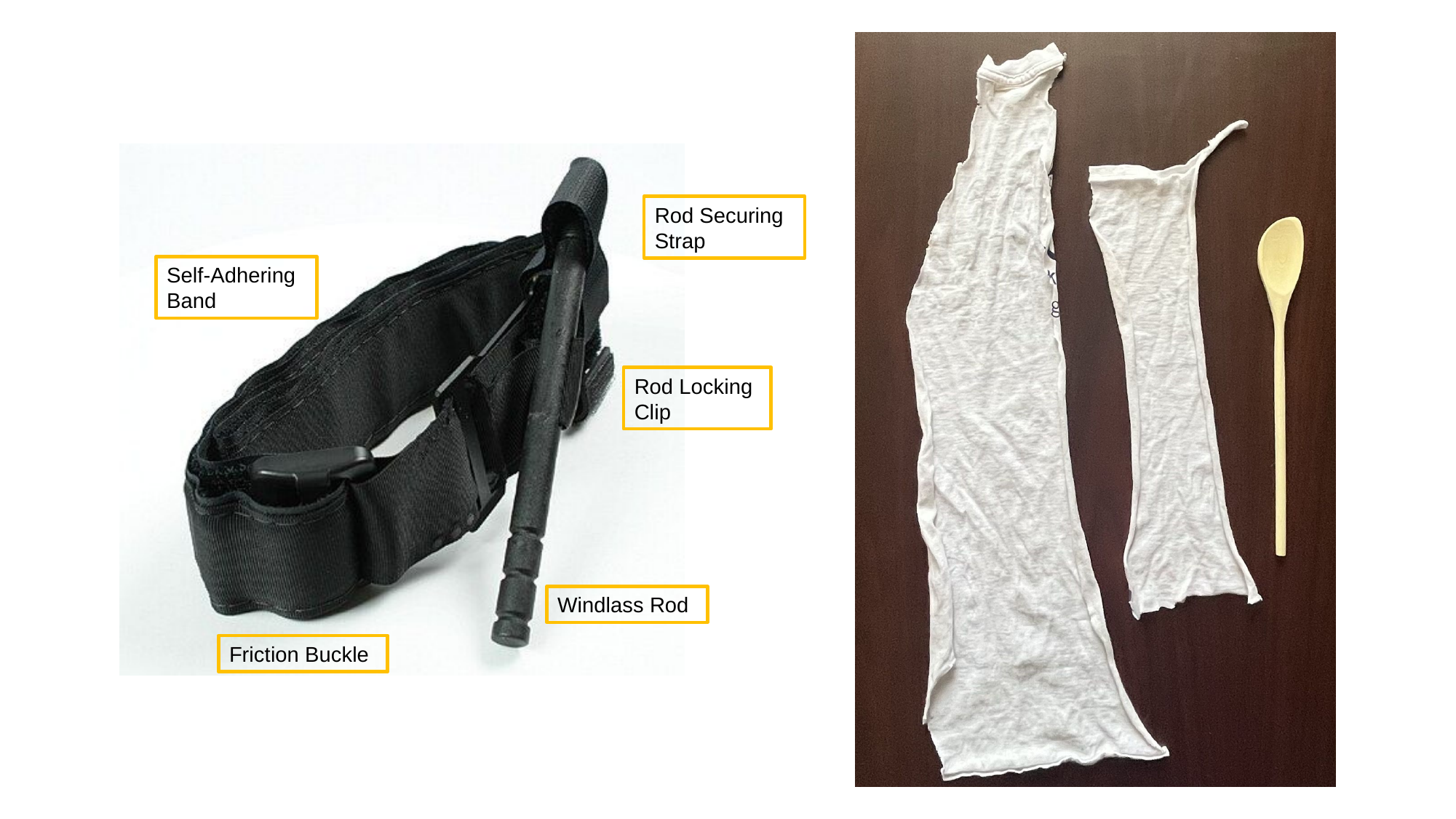

Rod Securing Strap
Self-Adhering Band
Rod Locking Clip
Windlass Rod
Friction Buckle

## Slide 17
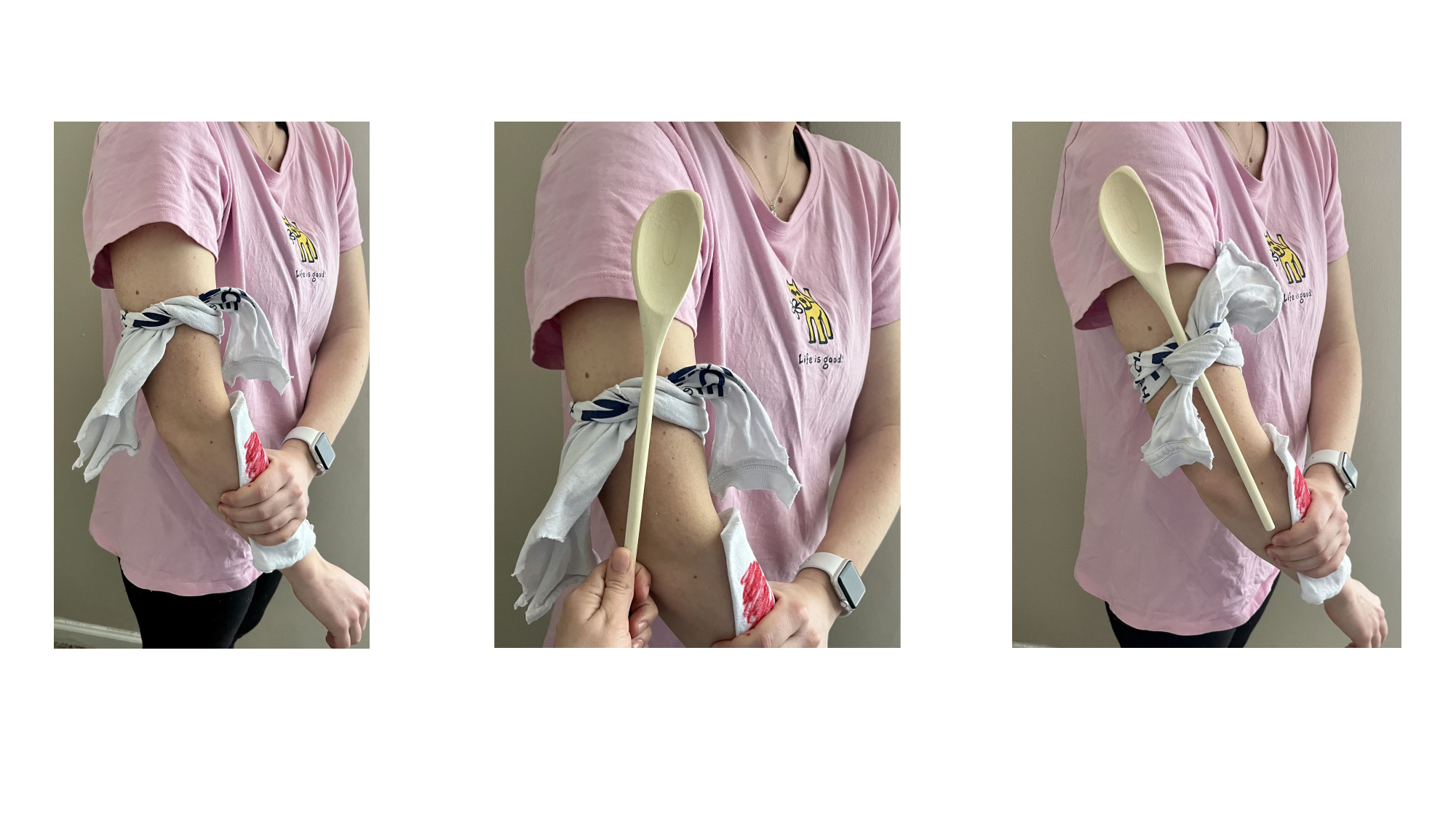

## Slide 18
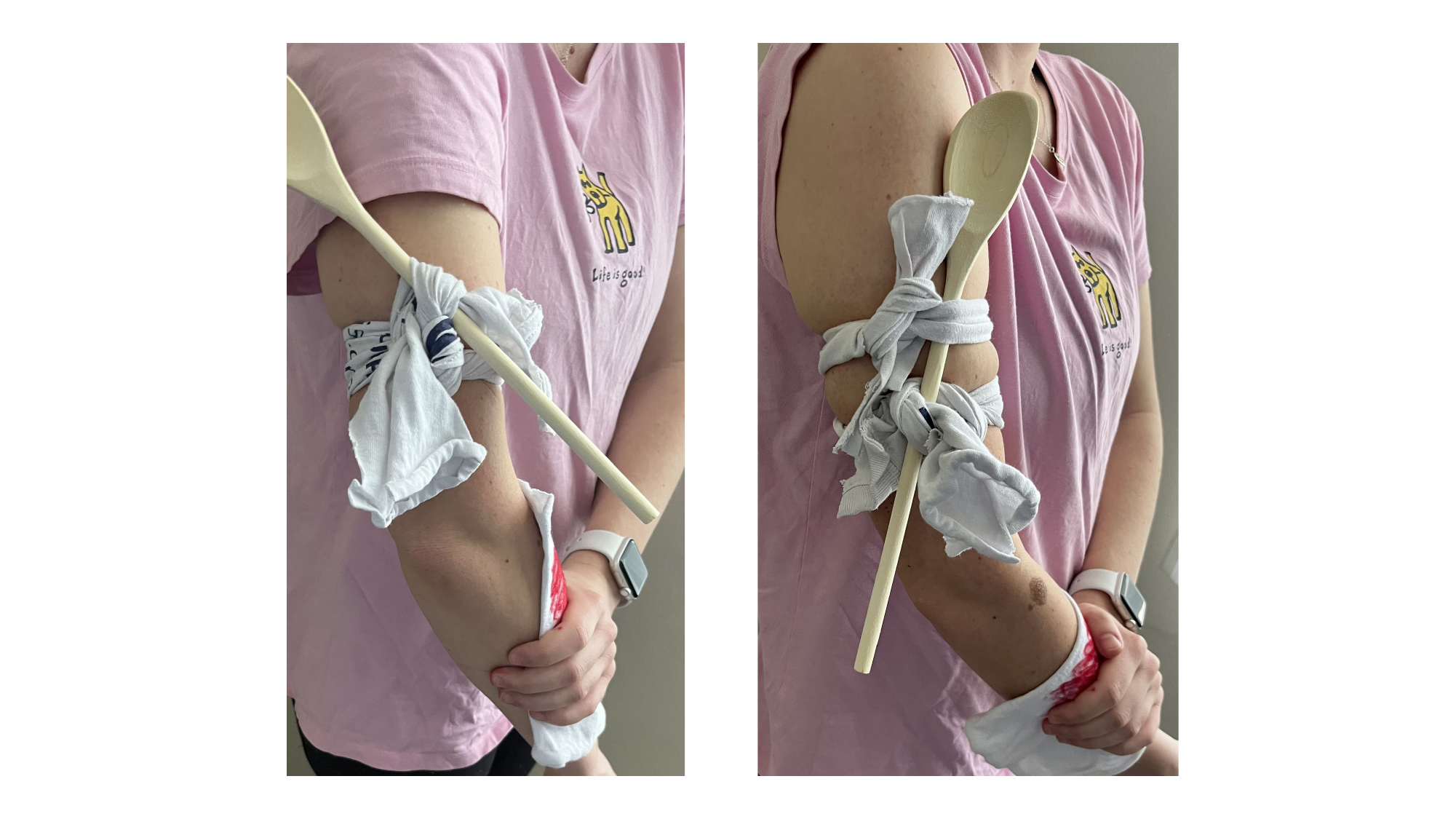

Supplement: Supplementary file 1 [file 9-3-SG63-AppendixB.pptx]
